# Characterization of Inducible Models of Tay-Sachs and Related Disease

**DOI:** 10.1371/journal.pgen.1002943

**Published:** 2012-09-20

**Authors:** Timothy J. Sargeant, Deborah J. Drage, Susan Wang, Apostolos A. Apostolakis, Timothy M. Cox, M. Begoña Cachón-González

**Affiliations:** 1 Department of Medicine, Addenbrooke's Hospital, University of Cambridge, Cambridge, United Kingdom; 2 Central Biomedical Services, School of Clinical Medicine, Addenbrooke's Hospital, University of Cambridge, Cambridge, United Kingdom; University College London, United Kingdom

## Abstract

Tay-Sachs and Sandhoff diseases are lethal inborn errors of acid β-N-acetylhexosaminidase activity, characterized by lysosomal storage of GM2 ganglioside and related glycoconjugates in the nervous system. The molecular events that lead to irreversible neuronal injury accompanied by gliosis are unknown; but gene transfer, when undertaken before neurological signs are manifest, effectively rescues the acute neurodegenerative illness in *Hexb−/−* (Sandhoff) mice that lack β-hexosaminidases A and B. To define determinants of therapeutic efficacy and establish a dynamic experimental platform to systematically investigate cellular pathogenesis of GM2 gangliosidosis, we generated two inducible experimental models. Reversible transgenic expression of β-hexosaminidase directed by two promoters, mouse *Hexb* and human *Synapsin 1* promoters, permitted progression of GM2 gangliosidosis in Sandhoff mice to be modified at pre-defined ages. A single auto-regulatory tetracycline-sensitive expression cassette controlled expression of transgenic *Hexb* in the brain of *Hexb−/−* mice and provided long-term rescue from the acute neuronopathic disorder, as well as the accompanying pathological storage of glycoconjugates and gliosis in most parts of the brain. Ultimately, late-onset brainstem and ventral spinal cord pathology occurred and was associated with increased tone in the limbs. Silencing transgenic *Hexb* expression in five-week-old mice induced stereotypic signs and progression of Sandhoff disease, including tremor, bradykinesia, and hind-limb paralysis. As in germline *Hexb−/−* mice, these neurodegenerative manifestations advanced rapidly, indicating that the pathogenesis and progression of GM2 gangliosidosis is not influenced by developmental events in the maturing nervous system.

## Author Summary

Sandhoff and Tay-Sachs disease are devastating neurological diseases associated with developmental regression, blindness, seizures, and death in infants and young children. These disorders are caused by mutations in β-hexosaminidase genes, which result in neuronal accumulation of certain lipids, glycosphingolipids, inside the lysosomes of neurons. It is not yet known how accumulation of lipids affects neuronal function, and although promising treatments such as gene therapy are in development, currently none has been clinically approved. We aimed to develop genetic models that allow manipulation of β-hexosaminidase expression over time. Two inducible strains of mice were created in which acute Sandhoff disease could be “turned on” by the addition of doxycycline in the diet. Once induced in the adult mouse, the disease progressed relentlessly and was apparently independent of the rapid developmental processes that occur in the fetal and neonatal brain, resembling disease course in the germline *Hexb−/−* mouse. These transgenic inducible strains of Sandhoff disease mice provide a dynamic platform with which to explore the pathophysiological sequelae immediately after loss of neuronal lysosomal β-hexosaminidase activity.

## Introduction

Two-thirds of the seventy or so inborn errors of lysosomal function affect the nervous system. Tay-Sachs disease [1], [2] and Sandhoff disease [3] are GM2 gangliosidoses arising from deficiency of the lysosomal acid hydrolase, β-N-acetylhexosaminidase; they are characterized by neuronal accumulation of GM2 ganglioside and related glycoconjugates [4]–[6]. Infantile GM2 gangliosidosis is a relentless neurodegenerative disorder with developmental regression, dystonia, blindness and seizures causing death in childhood [7], [8]. Characteristically, infants with GM2 gangliosidoses are healthy at birth and during the neonatal period but loss of motor function and cognition, with regression of acquired skills, becomes apparent after the first few months of life [9] - suggesting that disease onset is influenced by developmental processes involved in post-natal organization of the brain.

Development of genetically coherent models of Sandhoff disease generated by disruption of the *Hexb* gene in embryonic stem cells in mice [10], [11] provides a platform for pathological and therapeutic investigation of GM2 gangliosidoses. However, questions as to the pathogenesis, mechanisms inducing progression of disease, and the true extent of therapeutic reversibility remain. Ascertaining how the lysosomal defect contributes to widespread neuronal injury and other cardinal features of this condition, mandates the need for an authentic model of the disease which allows temporal and spatial dissection of the neuropathology to be analysed during its evolution. To accomplish this, we developed a reversible transgenic murine counterpart of human Sandhoff disease which utilizes the tetracycline-inducible gene expression system.

Mouse models employing the tetracycline-inducible system [12] have been created for the investigation of other neurogenetic diseases such as Huntington's disease [13] and Alzheimer's disease [14]. While these models used the tetracycline-inducible system to deliver a single deleterious gene product, creation of an informative experimental model to study diffuse neurodegeneration in a recessively transmitted disorder of lysosomal function, requires global rescue of the nervous system. Inherent challenges to this stratagem relate particularly to the extent of functional restitution and robustness with which long-term expression can be obtained in the neuraxis [15].

Here we characterize two novel inducible strains of transgenic Sandhoff disease mice: one expresses a construct harbouring proximal elements of the mouse *Hexb* promoter, its counterpart is under the control of the human *Synapsin 1* gene (*SYN1*) promoter. Phenotypic rescue of Sandhoff mice with autoregulatory expression cassettes based on the tet-off system led to a threefold extension of lifespan with sparing of most of the central nervous system from lysosomal storage and accompanying injurious effects. Unexpectedly, doxycycline-induced suppression of β-hexosaminidase expression in the adult animal caused acute neurodegeneration with the stereotypical murine simulacrum of Sandhoff disease and a course indistinguishable from the unmodified germline *Hexb−/−* background strain. Accordingly, neuro-developmental processes probably have little influence on the lysosomal metabolism of gangliosides or on the cellular ontogeny of Tay-Sachs and Sandhoff diseases. These findings may inform the timing and clinical stage at which therapeutic interventions such as gene therapy are considered for patients with GM2 gangliosidoses.

## Methods

### Ethics Statement

Mice were handled in accordance with the Animals (Scientific Procedures) Act 1986.

### Transgene Construction

Bovine growth hormone polyadenylation (BGH-polyA) sequence from the pcDNA3 plasmid (Invitrogen) was subcloned into the SphI site of pSP73 plasmid (P2221, Promega) to make plasmid P1. Coding sequence for the tet-transactivator from the pTet-off advanced plasmid (tTA2^s^, 630934, Clontech) was digested with EcoRI and BamHI and subcloned into the pSP73 plasmid upstream of the BGH-polyA sequence (P2). The proximal murine *Hexb* promoter (330 bp) [16] was amplified with primers: forward 5′-CTC CTG GGA ATT CTG ACT CG-3′ and reverse: 5′-TCC GCG AGT CTG GCT AGG-3′. The human *Synapsin 1* promoter [17] (*SYN1*, 585 bp) containing the neuron-restrictive silencing element [18] was amplified by PCR using primers: forward 5′-AGT CTT GTA CAC CCT CTG TGA GGG GGT TAT T-3′ and reverse 5′-AGT GTG AAT TCC TCT CAG GCA CGA CAC GAC-3′. Both promoter fragments were cloned using the TOPO 2.1 TA cloning vector (Invitrogen). Either one of these promoters was sequenced and subcloned into the EcoRI site upstream of the tet-transactivator in plasmid P2 after dephosphorylation of the vector with shrimp alkaline phosphatase (plasmids P3Hex or P3SYN).

The TRE-Tight promoter from pTRE-Tight (631059, Clontech) was digested with ZraI and EcoRI and subcloned into the EcoRV and EcoRI restriction sites upstream of the BGH-polyA sequence in P1 to make plasmid P4. The full murine *Hexb* cDNA was amplified from a plasmid (Image ID: 100015010, GenBank accession: BC146503) using primers: forward 5′-ATG CAG AAT TCA GCA GAA GGG CCG TCA AG-3′ and reverse 5′-CGA ACC GAA CAG GCT TAT GT-3′. This amplification product was cloned into a TOPO 2.1 plasmid and digested with EcoRI and StuI and subcloned into the EcoRI and EcoRV restriction sites of pcDNA3 for expression analysis. *Hexb* coding sequence was then digested with EcoRI and PvuII and subcloned into EcoRI and PvuII restriction sites downstream of the TRE-Tight promoter in plasmid P4 to make P5.

A BglII/BsrBI fragment containing the *Hexb* promoter/tTA-cDNA/BGH-PolyA construct from P3Hex and a BglII/HpaI fragment containing the *SYN1* promoter/tTA-cDNA/BGH-PolyA construct from P3SYN were subcloned into ZraI and BglII restriction sites in P5 such that they were in opposite ‘back-to-back’ orientation to the TRE-Tight/*Hexb*-cDNA/BGH-PolyA construct on the same vector. This created the auto-regulatory constructs P6Hex and P6SYN (Figure S1). The BGH-PolyA sequence downstream of the *Hexb* coding sequence in P6Hex and P6SYN was replaced with a NotI/PciI fragment from the pTRE-Tight plasmid containing SV40-PolyA sequence, creating plasmids P7Hex and P7SYN (Figure S1).

cHS4 sequence, reported to be a good insulating element [19], was amplified from genomic DNA isolated from commercially available chicken liver using primers: forward 5′-ACG TAG ATC TTC CTG GAA GGT CCT GGA AG-3′ and reverse 5′-TCA AAC ATG CAG GCT CAG AC-3′ and sequenced. Cloned cHS4 sequence was found to contain mutations when compared to published sequence. Consequently, this sequence was re-amplified using the forward primer 5′-ATA CGG AGA TCT GAG CTC ATG GGG ACA GCC CCC CCC CAA AGC CCC CAG GGA TGT AAT TAC-3′ in order to change the sequence of the second element of cHS4 so it was identical to sequence previously published. cHS4 was subcloned into a BglII site, in between the two expression constructs in each of the two P7 plasmids, to create four new plasmids where the cHS4 element was placed in either of two orientations – P8Hex (+), P8Hex (−), P8SYN (+) and P8SYN (−). P6Hex, P8Hex (+) and P8SYN (+) constructs were chosen for standard pronuclear injection techniques (Figure S1) [20].

### Generation of Transgenic Animals

P6Hex, P8Hex (+) and P8SYN (+) plasmids, digested with DrdI and PvuII to produce 3.8, 4.6, and 4.8 kbp fragments, respectively, were injected into fertilized oocytes produced from matings of B6CBA F1 mice. *Hex^Tg^* or *SYN^Tg^* transgenic founder animals, where Hexb cDNA was under the control of the *Hexb* or *SYN1* promoters, respectively, were genotyped by PCR. For routine genotyping, the presence of either the Hex or the SYN inducible constructs were detected using primers: 5′-AGC TCA CTC AAA GGC GGT AA-3′ and 5′-GGG AGG ATT GGG AAG ACA AT-3′ to amplify sequence across the tail to head junctions of the integrated tandem repeats.

Transgenic animals were crossed with germline *Hexb−/−* (Sandhoff) mice (strain: B6; 129S-*Hexb^tm1Rlp^*, Jackson Laboratory [10]). Crossing transgenic founder *Hex^Tg^* and *SYN^Tg^* mice and Sandhoff mice was performed until *Hexb−/−Hex^Tg^* or *Hexb−/−SYN^Tg^* mice were generated. The *Hex* and *SYN* transgenes were maintained in a hemizygous state. The presence or absence of the *Hexb* knockout allele was detected using a three primer PCR reaction: MNEO1: 5′-ATC TGG ACG AAG AGC ATC AG-3′; MHexb18: 5′-TAG ACT GCT TTG GAA ACT GC-3′; MHexb19: 5′-TCA GGA AGG AAG TGT CTC AC-3′.

### Animal Experimentation

Mice were allowed access to food ad libitum and were supplied with transgel (Charles River Laboratories) and mashed food pellets on the cage floor when they displayed signs of Sandhoff disease. For disease induction, mice were exposed continuously to doxycycline in food pellets at a dose of 1 g doxycycline/kg of food (S3949, Bio-Serv, NJ). Motor performance was assessed by the inverted screen test as previously described [21]. For biochemical studies, animals were culled by asphyxiation with CO_2_ and tissues rapidly dissected out and frozen on dry ice. For histological analysis, using fresh frozen tissues, animals were asphyxiated with CO_2_ and tissues embedded in OCT and frozen on dry ice. For histological analysis using perfuse-fixed tissue, animals were terminally anaesthetised with an overdose of sodium pentobarbital and transcardially perfused with phosphate-buffered saline (PBS) containing 4% w/v paraformaldehyde. Brains were postfixed for two hours in paraformaldehyde and cryopreserved in PBS containing 30% w/v sucrose.

### Histological Processing

OCT embedded tissue was sectioned at 15 or 30 µm thickness and mounted onto superfrost plus microscope slides, and stored at −80°C. For immunostaining, primary antibodies used were mouse anti-GFAP (1∶50, sc-6171, Santa-Cruz Biotechnology), rat anti-CD68 (1∶50, MCA-1957, Serotec), or rabbit anti-PAX2 (1∶100, 71–6000, Invitrogen). Secondary antibodies used were goat anti-mouse Ig-HRP (1∶100, P0477, DAKO), biotinylated anti-rat (1∶100, BA-4001, Vector Laboratories), or goat anti-rabbit Ig-HRP (1∶100, P0488, DAKO).

Slides were thawed, dried and fixed in PBS containing 4% paraformaldehyde for 10 minutes. Tissue was permeabilized in PBS containing 0.1% v/v Triton-X100 for 15 minutes and endogenous peroxidase activity was quenched by incubation in PBS containing 3% H_2_O_2_ for 5 minutes. Sections were blocked in PBS containing 2% bovine serum albumin for twenty minutes and were then incubated with blocking solution containing primary antibody overnight at 4°C. If the avidin/biotin complex system was used for staining, endogenous biotin was blocked at this stage using an avidin/biotin blocking kit (SP-2001, Vector Laboratories). Sections were washed in PBS three times for five minutes each and incubated in blocking solution containing secondary antibody for one hour. Sections that were incubated with biotinylated anti-rat antibody were washed in PBS as above and incubated with avidin-HRP (Vectastain ABC kit, PK-4000, Vector Laboratories) according to manufacturer's directions. Slides were washed in PBS as above and developed with diaminobenzidine (DAB, SK-4100, Vector Laboratories), dehydrated, cleared and mounted in DPX.

Detection of β-hexosaminidase activity in tissues was carried out as previously described [22] by incubating sections in the substrate naphthol AS-BI N-acetyl-β-glucosaminide (Sigma, N4006-1G). Hex-azotized pararosaniline, prepared by treatment of pararosaniline (P3750-5G, Sigma) with sodium nitrite and hydrochloric acid, was used to visualise the enzymatic reaction-product. For co-localization with the neuron-specific marker, NeuN, sections stained for β-hexosaminidase activity were permeabilized and blocked as above, and then subsequently incubated with mouse anti-NeuN-Alexa 488 conjugate (1∶100, MAB377X, Millipore) for one hour. Slides were washed in PBS and mounted in Prolong Gold anti-fade mounting medium (P36931, Invitrogen). Periodic acid-Schiff (PAS) staining was carried out with Schiff reagent (3803800E, Leica Microsystems) and periodic acid (P0430-100G, Sigma) as instructed by the manufacturer. PAS-stained sections were counterstained with haematoxylin, dehydrated, cleared and mounted in DPX.

In situ hybridization was performed as previously described [23]. Expression of tet-transactivator mRNA was assessed by hybridization with an antisense probe generated using the T7 promoter on plasmid P2 that was cut with EcoRI (1.1 kb).

### Tissue Analysis

Fluorometric analysis of enzyme activity using the substrate 4-methylumbelliferyl-2-acetamido-2-deoxy-β-D-glucopyranoside (MUG, M-5504, Biosynth), was performed as previously described [23]. Briefly, tissue samples were homogenized in 10 mM sodium phosphate (pH 6.0) containing 100 mM NaCl and 0.1% Triton X-100, centrifuged at 13,000 RPM for 10 minutes and the supernatant taken for enzyme analysis. Activities were standardized by determination of protein concentration using the bicinchoninic acid protein assay (23227, Thermo Scientific). Anion-exchange chromatography, used to resolve the activity of the different β-hexosaminidase isoforms (HexA, HexB and HexS), was carried out as described previously [23].

Lipids were quantified using high performance thin–layer silica chromatography as previously described [21]. Relative quantification by densitometry was performed using ImageJ software (NIH) and sphingolipids were standardized to ganglioside GM1.

Gene expression was quantified by real-time PCR. Mouse tissue was homogenised and total RNA extracted using an RNeasy Mini Kit (74104, Qiagen). Total RNA was reverse-transcribed with a Quantitect Reverse Transcriptase Kit (205311, Qiagen). Real-time PCR was performed with Power SYBR Green PCR Master Mix (4367659, Applied Biosystems) on a 7500 Fast Real-Time PCR System (Applied Biosystems). Gene expression was standardized by comparison with the housekeeping gene for β-actin, using the commercially available primer set Quantitect Primer Assay Mm_Actb_2_SG (QT01136772, Qiagen). Transgenic mouse *Hexb* cDNA was amplified using the forward and reverse primers: 5′- AAT GGT CAG CCG TGG AAT AG-3′ and 5′- CAA ATG TGG TAT GGC TGA TTA TG-3′. Transgenic tet-transactivator (tTA2^2^) cDNA sequence was amplified using the forward and reverse primers: 5′- AGA GCA CAG CGG AAT GAC TT-3′ and 5′- CCT GTA CTG GCA CGT GAA GA-3′. For all amplification reactions, the following cycle conditions were used: 95°C for 10 min×1; 95°C for 15 s, 58°C for 1 min×35.

### Image Manipulation, Counting, and Statistical Analysis

All drawing was performed using Inkscape (version 0.47). Manipulation of photomicrographs was carried out with ImageJ (NIH) and arranged using Inkscape.

PAX2-positive neurons in the ventral spinal cord were quantified using ImageJ (NIH) software. For each of the four regions of the spinal cord counted, three non-consecutive 15 µm thick sections were quantified per animal. PAX2-stained neurons ventral to the central canal were counted. Counts were standardized by area to give a relative density per mm^2^. For statistical analysis, ANOVA was used with Bonferroni's post hoc analysis for comparison (GraphPad Prism v5.0, GraphPad Software).

## Results

### Assessment of Transgenic β-Hexosaminidase Expression

**Figure 1:**
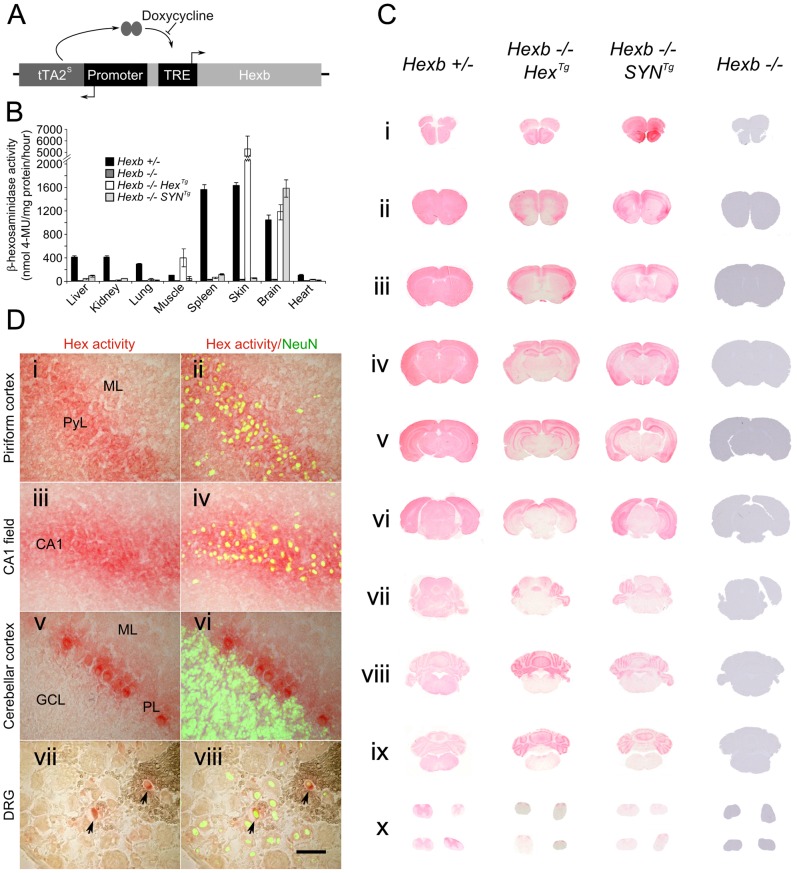
Expression of *Hexb* from two inducible constructs throughout the Sandhoff mouse neuraxis. (A) Expression of transgenic *Hexb* from a single autoregulatory cassette was driven by either the mouse *Hexb* promoter (Hex line) or the human *SYN1* promoter (SYN line). Tet-transactivator expressed from tTA2^s^ coding sequence promoted expression of *Hexb* cDNA from tet-response elements (TRE). This was inhibited by doxycycline. (B) Expression of transgenic *Hexb* in different tissues was assessed with the MUG assay. β-hexosaminidase activity was found in the brain for both Hex and SYN lines, but also in the skin and skeletal muscle for the transgenic Hex mouse line. Bars = mean ± SEM. n = 3. Panel C shows expression of β-hexosaminidase activity in *Hexb−/−Hex^Tg^* and *Hexb−/−SYN^Tg^* mice assessed by staining using the enzyme substrate naphthol AS-BI N-acetyl-β-glucosaminide (red staining). Strong expression of β-hexosaminidase activity is seen in the cortex (C i–vi) and the cerebellum (C vii–ix) of both lines. Weaker expression is seen in the diencephalon in the SYN line (C iv–v) while activity is very weak to absent in the mid (C vi–vii) and hindbrain (C vii–ix). (D) β-hexosaminidase activity staining was associated with neurons, shown by co-labelling with NeuN (green fluorescence) in the piriform cortex (i and ii), CA1 field of Ammon's horn (iii and iv), the cerebellar cortex (v and vi) and the dorsal root ganglia (vii and viii), where only some neurons expressed transgenic β-hexosaminidase activity. Images represent staining from *Hexb−/−Hex^Tg^* mice (DRG and cerebellar cortex) and *Hexb−/−SYN^Tg^* mice (CA1 field and piriform cortex). ML = molecular layer, PyL = pyramidal layer, CA1 = CA1 field, PL = Purkinje neuron layer, GCL = granule cell layer. Scale bar = 50 µm.

Inducible expression constructs were tested by transient transfection of HEK 293T cells, with and without exposure to various concentrations of doxycycline. Two main types of inducible cassette were tested – one expressing from the proximal mouse *Hexb* promoter (Hex), the other from the human *SYN1* promoter (SYN). Both inducible constructs showed strong expression of β-hexosaminidase activity, and brisk silencing in response to low concentrations of doxycycline added to the media of transiently transfected cells (Figures S1 and Figure 1A). Constructs were then microinjected into fertilized oocytes to generate transgenic founder mice (Table S1). Constructs driven from both the *Hexb* and *SYN1* promoters were used to create transgenic lines that were intended to compare body-wide rescue from Sandhoff disease with rescue in nervous tissues only.

Transgenic founder mice were identified using PCR and crossed over two successive generations onto a *Hexb−/−* background. Two transgenic lines, one bearing a Hex cassette (*Hexb−/−Hex^Tg^*) and one bearing a SYN cassette (*Hexb−/−SYN^Tg^*) (from P8Hex (+) and P8SYN (+), respectively), expressed transgenic β-hexosaminidase in the brain, as shown by a fluorometric MUG assay performed on homogenized brain tissue (Figure 1B). As anticipated, transgenic β-hexosaminidase expression from the *SYN1* promoter was found only in nervous tissues. Contrary to expectation, the Hex cassette did not express in the wide variety of tissues as the endogenous mouse *Hexb* promoter does. Instead, expression was found only in the nervous tissue, skin and skeletal muscle.

Anion-exchange chromatography on tissue lysate from the cerebrum showed the presence of all three β-hexosaminidase isozymes, HexB, HexA and HexS, in the brains of both lines; the presence of HexB and HexA rely on expression of transgenic *Hexb* coding sequence (Figure S2C and S2E). Control heterozygous (*Hexb+/−*) and germline mutant (*Hexb−/−*) animals showed the predicted pattern of β-hexosaminidase expression. Importantly, the relative amounts of the three isozymes in the transgenic lines and heterozygous mice were similar.

Throughout the neuraxis, transgenic β-hexosaminidase expression, assessed by staining for β-hexosaminidase activity on cryo-sections, appeared to be more widespread in the *Hexb−/−SYN^Tg^* line than in the *Hexb−/−Hex^Tg^* strain. The SYN transgenic mouse expressed β-hexosaminidase activity throughout the forebrain, including the diencephalon where activity was largely weak or absent in the Hex line (Figure 1C iii–v). In both lines transgenic β-hexosaminidase activity was present in the cerebellum and absent in the brainstem, with a few exceptions in the Hex line; the inferior colliculi and the inferior olivary complex did show enzyme activity expression. Interestingly, strong transgene expression was associated with neurons, rather than with glia in both lines (Figure 1D).

The pattern of β-hexosaminidase staining throughout the neuraxis showed that expression appeared restricted to particular neuronal populations. β-hexosaminidase can be secreted and recaptured by neighbouring cells [24]. To define the pattern of neuronal cell populations expressing the transgene and potential relevance to disease manifestations, in situ hybridization was used against the tet-transactivator. Strong expression of tet-transactivator transcript was observed in the brains of mice carrying *SYN^Tg^* or *Hex^Tg^* transgenes, with a few notable differences. In the *SYN^Tg^* cerebral cortex, tet-transactivator transcript was strongly expressed in layer 6b neurons, but not as strongly expressed in other layers. This contrasted with the Hex line, where expression was more evenly distributed through the cortex. Furthermore, strong hybridization signal found in the olfactory bulbs and the thalamic reticular nucleus of the Hex transgenic animal was absent in the SYN line (Figure S3). It is noteworthy that even though no in situ hybridization signal could be seen in the *SYN^Tg^* strain olfactory bulbs, there was strong transgenic β-hexosaminidase activity. However, in most cases expression of tet-transactivator transcript was seen in the same populations of cells that strongly expressed β-hexosaminidase activity.

### Inducible Hexb Expressing Transgenes Rescue the Sandhoff Mouse

**Figure 2:**
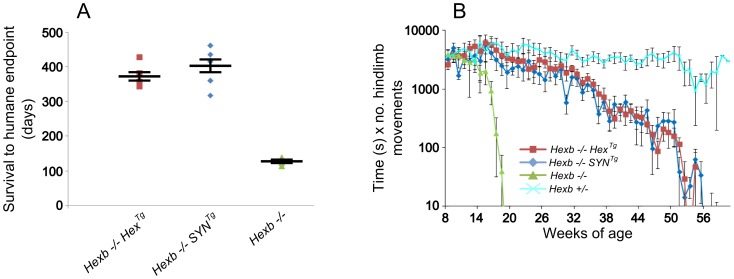
Inducible transgenic constructs rescue mice from Sandhoff disease. (A) *Hexb−/−* mice that carry the Hex or SYN transgenic cassettes show an average survival of 373 or 404 days, respectively. This is a three-fold increase on *Hexb−/−* mice that do not carry a transgenic expression construct and only survive to an average of 127 days. Plots show data points overlaid with the mean ± SEM. (B) Motor performance of transgenic animals was assessed using the inverted screen test and performance measured by multiplying latency to fall (seconds) by number of hindlimb movements. *Hexb−/−* Sandhoff mice rapidly deteriorated after 14 weeks of age (green triangles). *Hexb−/−Hex^Tg^* and *Hexb−/−SYN^Tg^* mice showed motor performance comparable with *Hexb+/−* mice up until six months of age, by which point transgenic mice began progressive deterioration that culminated in humane endpoint at approximately one year of age. n = 6, 8, 11 and 9 mice for *Hexb−/−Hex^Tg^*, *Hexb−/−SYN^Tg^*, *Hexb−/−* and *Hexb*+/− respectively. Data points represent mean ± SEM.

Expression of β-hexosaminidase activity from the Hex and SYN transgenic cassettes was sufficient to rescue the Sandhoff mouse from stereotypic features of acute Sandhoff disease. By the time the *Hexb−/−* Sandhoff animals reach four to five months of age, they show marked bradykinesia and tremor when compared with heterozygous mice of the same age (Videos S1 and S2). By this stage the Sandhoff mouse loses weight and reaches its humane endpoint (defined as losing 10–20% of its highest body weight) at an average age of 127 days. In contrast, Sandhoff mice that carry either the Hex or SYN transgenic constructs show unrestricted movement and no overt disease up to six months of age (Videos S3 and S4, respectively). However, the transgenic constructs do not provide complete rescue, and mice deteriorate between six months of age and their humane endpoints that are on average 373 days and 404 days for *Hexb−/−Hex^Tg^* and *Hexb−/−SYN^Tg^* mice, respectively (Figure 2A).

*Hexb−/−Hex^Tg^* and *Hexb−/−SYN^Tg^* mice show progressive but comparatively milder tremor than their germline counterparts that is accompanied by progressive limb hypertonia, evident when mice are held by their tails (Videos S5 and S6). By approximately one year of age, limb hypertonicity restricted movement of the mouse around its home cage; during walking the hindlimbs appeared un-coordinated (particularly evident in the mouse shown in Video S6). Hypertonicity in the limbs is a major contributing factor to deterioration in motor performance as measured with the inverted screen test, between six months and one year of age (Figure 2B).

**Figure 3:**
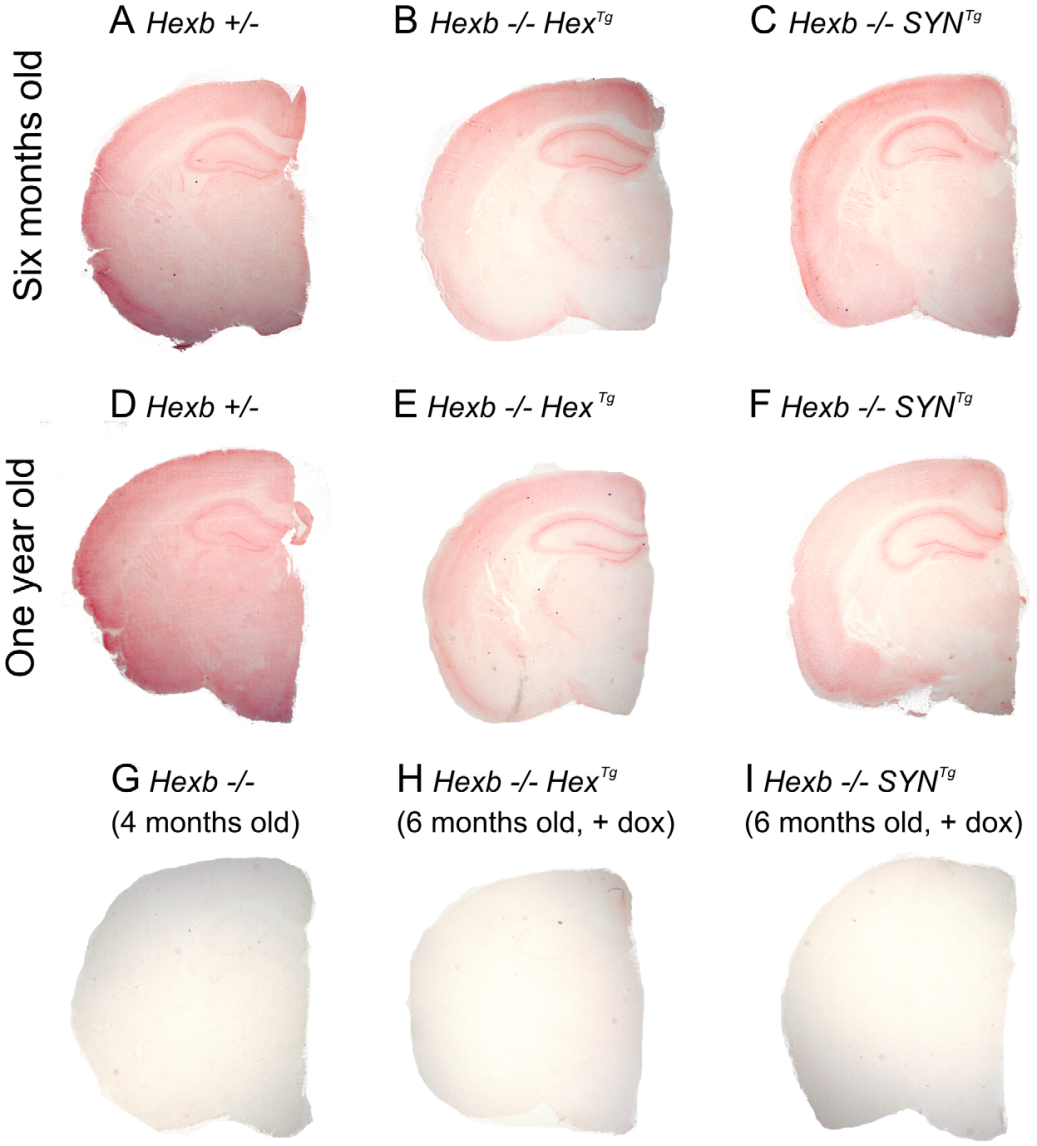
Pattern of β-hexosaminidase activity staining in transgenic mouse brain. Staining for β-hexosaminidase activity (red) was performed on 30 µm cryo-sections of mouse cerebrum. Controls are *Hexb+/−* (A) and *Hexb−/−* (G). Both *Hexb−/−Hex^Tg^* and *Hexb−/−SYN^Tg^* brains show staining for β-hexosaminidase activity in the absence of doxycycline (B and C). At one year of age, both Hex and SYN transgenic lines still show stable transgene expression (E and F). Expression of activity is completely repressed in the presence of doxycycline (H and I).

**Figure 4:**
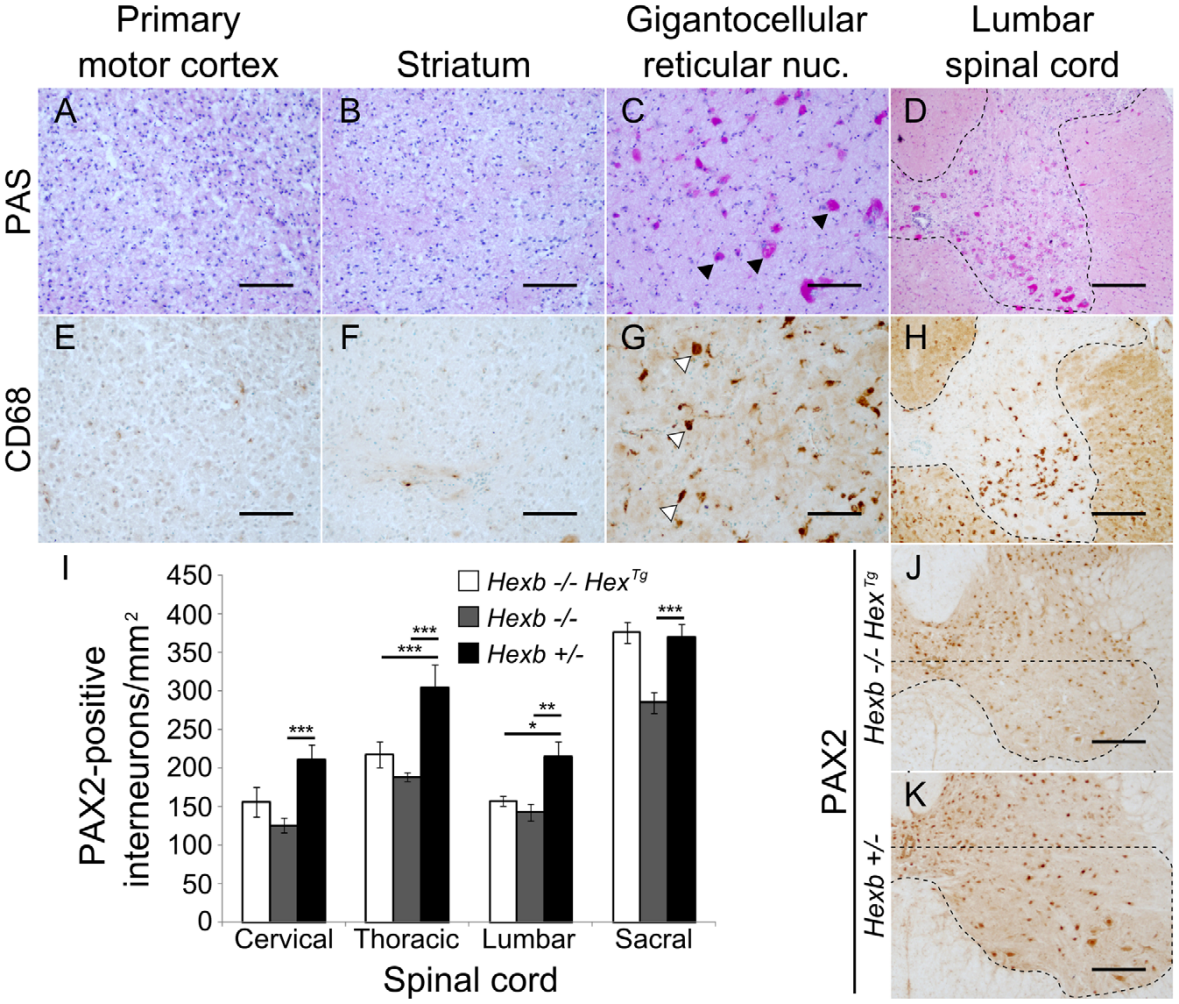
Localized glycolipid storage and microgliosis in *Hexb−/−Hex^Tg^* mice at humane endpoint. PAS stained brain sections show regions of the cerebrum such as the primary motor cortex (A) and the striatum (B) are devoid of glycolipid storage that stains magenta. However, storage is a prominent feature in the hindbrain of the same animals. C and D show glycolipid storage in neurons of the brainstem (gigantocellular reticular nucleus) and in the spinal cord grey matter respectively (C, arrowheads; D, dashed line). (E–H) Staining for activated microglia is revealed by brown DAB staining for CD68 and coincides with storage (G, arrowheads show CD68 staining microglia; H, dashed line shows spinal grey matter). (I) PAX2-positive ventral horn interneurons were quantified for *Hexb−/−Hex^Tg^*, *Hexb−/−* (both humane endpoint) and *Hexb+/−* (one year old) animals (n = 6, 8 and 6. Bars = mean ± SEM. *, P<0.05; **, P<0.01; ***, P<0.001 – Bonferroni post hoc test). Both *Hexb−/−Hex^Tg^* and *Hexb−/−* animals showed loss of PAX2-positive neuron density in multiple regions of the ventral spinal cord compared with *Hexb+/−* animals. J and K show PAX2 stained lumbar spinal cord used for quantification. The dashed line encompasses the region quantified. Scale bars: A–C and E–G = 50 µm; D, H, J and K = 100 µm.

Transgenic *Hexb* expression is still present in animals at their humane endpoint and is comparable with mice that are six months of age (Figure 3B–3C and 3E–3F). This is also reflected by the fact that when transgenic animals are culled, areas that strongly express transgenic *Hexb*, such as the cerebral cortex and the cerebellum, remain free of glycolipid storage as determined by PAS stained cryo-sections (Figure S4, Table S2). Overall, *Hexb−/−Hex^Tg^* and *Hexb−/−SYN^Tg^* mice show no storage in the cerebral cortex (including the motor corticies, Figure 4A) and most other structures in the cerebrum, such as the striatum (Figure 4B), beyond one year of age (humane endpoint). However, *Hexb−/−SYN^Tg^* mice did show less neuronal storage in the hypothalamic, midbrain and brainstem structures than *Hexb−/−Hex^Tg^* mouse line at humane endpoint (Table S2).

In Sandhoff animals rescued with a transgenic expression construct, nuclei of the hindbrain such as the gigantocellular reticular nucleus showed neurons that had substantial glycoconjugate storage (Figure 4C). Both *Hexb−/−Hex^Tg^* and *Hexb−/−SYN^Tg^* mice accumulated extensive amounts of storage material in the hindbrain and ventral spinal cord by one year of age (Figure 4C and 4D), accompanied by activated microglia (Figure 4G and 4H). Injury to these lower motor nuclei may account for the marked hypertonicity seen in the transgenic animals older than six months (Videos S5 and S6). To further characterize pathological processes in the ventral spinal cord of the Hex line at humane endpoint where abundant amoeboid microglia were seen, ventral PAX2-positive interneurons were counted (Figure 4I, 4J and 4K). Compared with one year old *Hexb+/−* mice, *Hexb−/−* and *Hexb−/−Hex^Tg^* mice at their respective humane endpoints had decreased PAX2-positive interneuron density in their ventral horns. Interestingly, loss of PAX2-positive interneuron density in *Hexb−/−* and *Hexb−/−Hex^Tg^* mice did not differ significantly in regions above the sacral spinal cord and also did not co-vary with degrees of hindlimb spasticity observed at the humane endpoint (Videos S2 and S5).

Variegation of transgene expression occurred in some regions of the brain such as the dorsal root ganglia (Figure 1D vii–viii). In some cases, neurons with abundant intense PAS-stained material were present in apposition to similar neurons without any apparent storage (not shown). Similar examples indicating a lack of complete cross-complementation were detected in numerous other nuclei of the mid and hindbrain in *Hexb−/−Hex^Tg^* and *Hexb−/−SYN^Tg^* mice.

### Transgenic Hexb Expression Is Reversibly Inducible In Vivo

**Figure 5:**
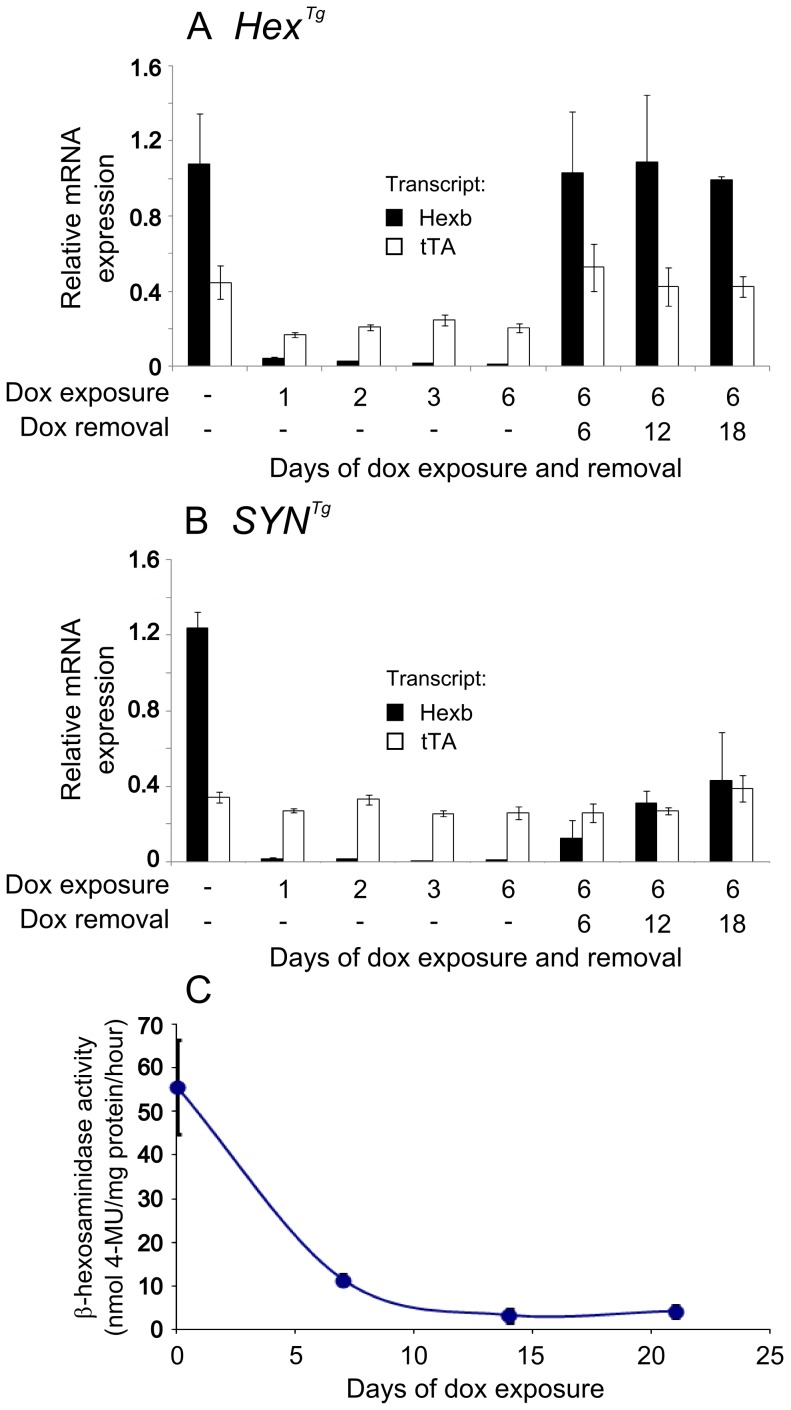
Inducible expression of transgenic constructs in the brain. Relative mRNA expression was analysed in mouse forebrain using primers specific for transgenic *Hexb* (black bars) and tet-transactivator (white bars), standardized to β-actin transcript. Animals used for analysis of transgene expression were *Hexb+/−Hex^Tg^* or *Hexb+/−SYN^Tg^*. Panel A shows expression analysis of mice bearing the Hex construct. When no doxycycline is present, transgenic *Hexb* exceeds tet-transactivator expression. Within one day of doxycycline exposure, *Hexb* expression is almost completely repressed. When doxycycline is removed, *Hexb* expression returns within six days and is stable thereafter. In SYN transgenic animals (B), suppression of transgenic *Hexb* with doxycycline resembled the Hex line. In contrast, when doxycycline was removed, transgenic *Hexb* recovered more slowly. Bars represent mean ± SEM. n = 3 per time point except the first timepoint of each A and B where n = 4. (C) Total β-hexosaminidase activity in brain lysates was measured by MUG cleavage at timepoints post doxycycline exposure to determine how long transgenic *Hexb* protein lasted in the Sandhoff mouse brain. When *Hexb−/−SYN^Tg^* animals were exposed to doxycycline, low levels of β-hexosaminidase activity could still be seen one week later, and reached its minimum by two weeks of doxycycline exposure. Data points = mean ± SEM. n = 3 animals per timepoint.

Transgene expression in mouse cerebrum was assessed with quantitative real-time PCR. Expression of both transgenic *Hexb* transcript and tet-transactivator transcript from the single autoregulatory cassette was standardized by comparison to endogenous β-actin transcript. In the absence of doxycycline, *Hexb* expression was greater than tet-transactivator expression. After only 24 hours of exposure to doxycycline, transgenic *Hexb* expression in the cerebrum had decreased by 96% and 98% in *Hex^Tg^* and *SYN^Tg^* mice respectively (Figure 5A and 5B). This reduction was stable over the next six days of doxycycline exposure.

To determine whether transgenic *Hexb* expression could return after being repressed, animals were fed doxycycline laced food (1 g doxycycline/kg food) for six days and sacrificed six, 12 and 18 days post doxycycline withdrawal (Figure 5A and 5B). *Hex^Tg^* mice showed rapid recovery of transgenic *Hexb* expression, within six days. Transgenic *Hexb* expression post doxycycline withdrawal in the forebrain was approximately equal to pre-doxycycline exposure levels and did not change between six and 18 days post doxycycline withdrawal (Figure 5A). In contrast, transgenic *Hexb* expression in *SYN^Tg^* mice barely increased at six days after doxycycline withdrawal. Transgenic *Hexb* expression recovered progressively over the next 12 days but did not reach pre-doxycycline exposure levels after 18 days withdrawal.

In line with a reduction in transgenic *Hexb* mRNA on exposure to doxycycline, β-hexosaminidase activity also decreased after transgenic animals were exposed to doxycycline. β-hexosaminidase activity was measured in brain lysates (*Hexb−/−SYN^Tg^* mice) using the MUG assay after 0, 7, 14 and 21 days of exposure to doxycycline. Half life of *Hexb* protein was approximately four days in the mouse brain and activity had dropped to its minimum within 14 days of doxycycline exposure (Figure 5C).

### Induction of Sandhoff Disease in the Adult Mouse Results in Stereotypic Sandhoff Disease

Once we established that the Hex and SYN transgenic cassettes provide rescue from acute Sandhoff disease, and were sensitive to doxycycline treatment in vivo, mice were fed doxycycline (1 g/kg of food) to continuously suppress β-hexosaminidase expression from five weeks of age until they reached their humane endpoint. This experiment tested whether suppression of transgenic *Hexb* expression permitted the accumulation of glycosphingolipids and at the same time, addressed the question as to whether developmental factors interacted with the course and signs of disease.

**Figure 6:**
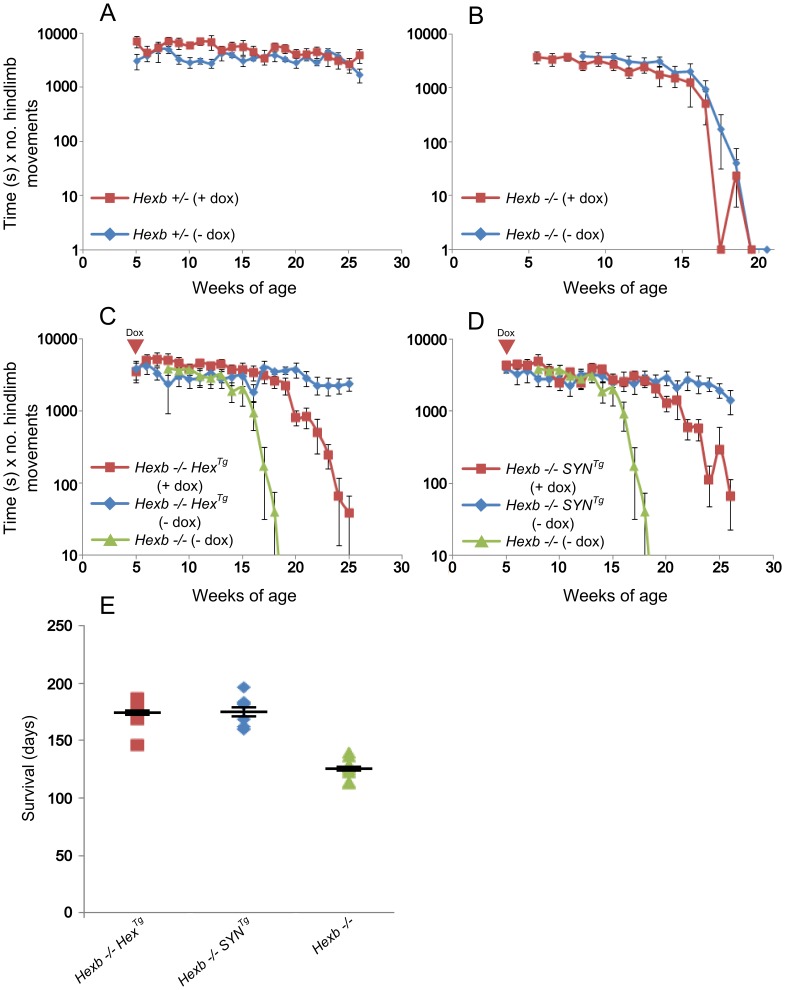
Suppression of *Hexb* expression results in development of stereotypic Sandhoff disease. A to D show motor performance measured by the inverted screen test. (A) No difference exists between two groups of healthy control mice (*Hexb+/−*); with (red, n = 5) and without (blue, n = 6) doxycycline treatment starting at five weeks of age. To determine if doxycycline itself modified Sandhoff disease (B), *Hexb−/−* mice were also maintained with (red, n = 6) and without (blue, n = 10) doxycycline. (C) *Hexb−/−Hex^Tg^* mice maintained steady performance on the inverted screen test (blue, n = 6). In contrast, when *Hexb−/−Hex^Tg^* mice were exposed to doxycycline from five weeks of age, their performance began to deteriorate from about 20 weeks of age onward (red, n = 8). This rapid deterioration in performance mimics that of *Hexb−/−* mice (green, n = 10). (D) Similar results were obtained for *Hexb−/−SYN^Tg^* mice with (red, n = 8) and without (blue, n = 8) doxycycline. Data points represent mean ± SEM. E shows survival of *Hexb−/−Hex^Tg^* and *Hexb−/−SYN^Tg^* mice exposed to doxycycline from five weeks of age (mean = 172.5 days, n = 8, and 175 days, n = 8, respectively). Survival of germline *Hexb−/−* mice is on average 127 days of age (n = 11), similar to the length of time inducible mice survive under doxycycline mediated suppression of transgenic *Hexb*. Plots show data points overlaid with the mean ± SEM.

Analysis of motor performance using the inverted screen test showed that the doxycycline treatment regimen did not impact on the motor performance of *Hexb+/−* animals (Figure 6A), nor did it alter the disease course of *Hexb−/−* animals (Figure 6B). As shown in Figure 6C and 6D, *Hexb−/−Hex^Tg^* and *Hexb−/−SYN^Tg^* animals that were not exposed to doxycycline showed stable motor performance over the time tested. However, animals of the same age and genotype that were fed doxycycline from five weeks of age showed a dramatic decrease in motor performance starting at age 20 weeks. Reduced motor performance of *Hexb−/−* animals began at 15 weeks.

*Hexb−/−Hex^Tg^* and *Hexb−/−SYN^Tg^* mice that were fed doxycycline from five weeks of age onwards developed progressive tremor from 17–19 weeks of age. Mouse weight, on average, had reached a plateau by 20 weeks of age and thereafter started to decrease (Figure S5). Mice reached their humane endpoints with stereotypic acute Sandhoff disease on average 172.5 and 175 days of age, respectively (Figure 6E). Survival of *Hexb−/−Hex^Tg^* and *Hexb−/−SYN^Tg^* animals under exposure to doxycycline from five weeks of age was similar to total survival of germline *Hexb−/−* animals. We conclude that induction of acute Sandhoff disease in the adult mouse does not modify signs and disease course significantly. At the humane endpoint, these mice showed typical features of acute Sandhoff disease, such as bradykinesia and tremor (Videos S7 and S8), unlike mice of the same genotype and age that had not been exposed to doxycycline (Videos S3 and S4).

Staining for β-hexosaminidase activity in the brains of *Hexb−/−Hex^Tg^* and *Hexb−/−SYN^Tg^* mice that were exposed to doxycycline until their humane endpoint (Figure 3H and 3I), showed complete suppression of *Hexb* expression throughout the entire neuraxis. Similarly, anion-exchange chromatography performed on samples of cerebrum showed loss of HexB and HexA β-hexosaminidase isoforms upon doxycycline exposure (Figure S2D and S2F).

**Figure 7:**
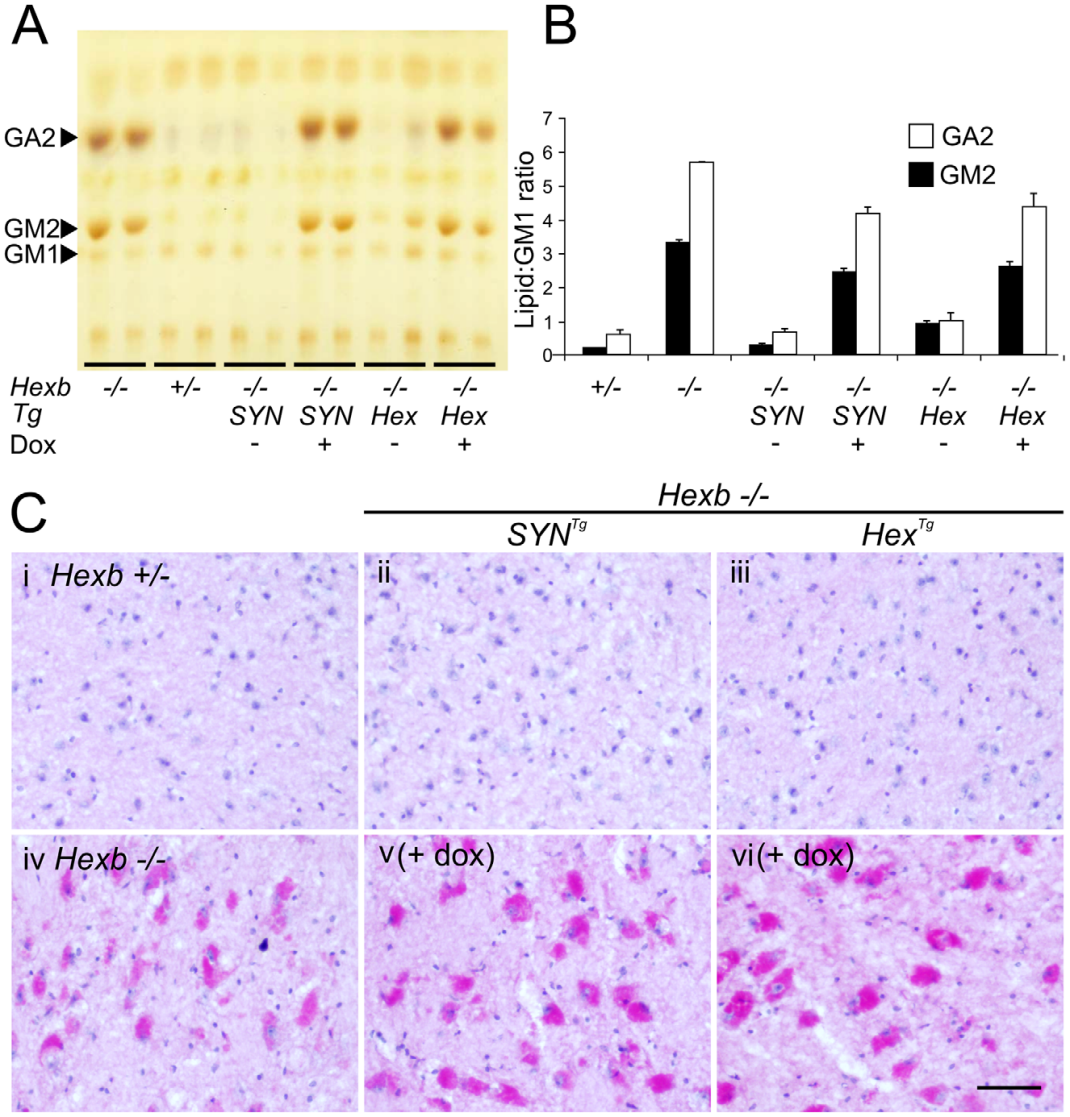
Doxycycline mediated silencing of transgenic *Hexb* expression induces storage of glycolipids. (A and B) Thin layer chromatography shows increase in the amount of GA2 and GM2 lipid in extracts of Sandhoff mouse cerebrum that were taken at the humane endpoint. Only trace amounts of the same lipids exist in age-matched heterozygous controls. Both SYN and Hex transgenic constructs prevented the accumulation of GM2 and GA2 in the Sandhoff mouse at approximately six months of age. When *Hexb−/−Hex^Tg^* or *Hexb−/−SYN^Tg^* mice were fed doxycycline from five weeks of age until their humane endpoint, these lipids accumulated to amounts seen in the Sandhoff mouse at humane endpoint (n = 4, 2, 3, 3, 4, 4, for *Hexb+/−*, *Hexb−/−*, *Hexb−/−SYN^Tg^* (−Dox) and (+Dox), *Hexb−/−Hex^Tg^* (−Dox) and (+Dox), respectively). (C) PAS staining in the thalamus shows weak staining in the *Hexb+/−* animal (i) and strong staining in neurons of the Sandhoff animal at humane endpoint (iv, magenta staining). *Hexb−/−Hex^Tg^* and *Hexb−/−SYN^Tg^* animals were protected from accumulation of lipids in the thalamus, shown by a lack of PAS staining (ii and iii). In animals that were fed doxycycline, PAS staining revealed significant accumulation of glycoconjugates (v and vi). Sections were counterstained with haematoxylin. Scale bar = 50 µm.

**Figure 8:**
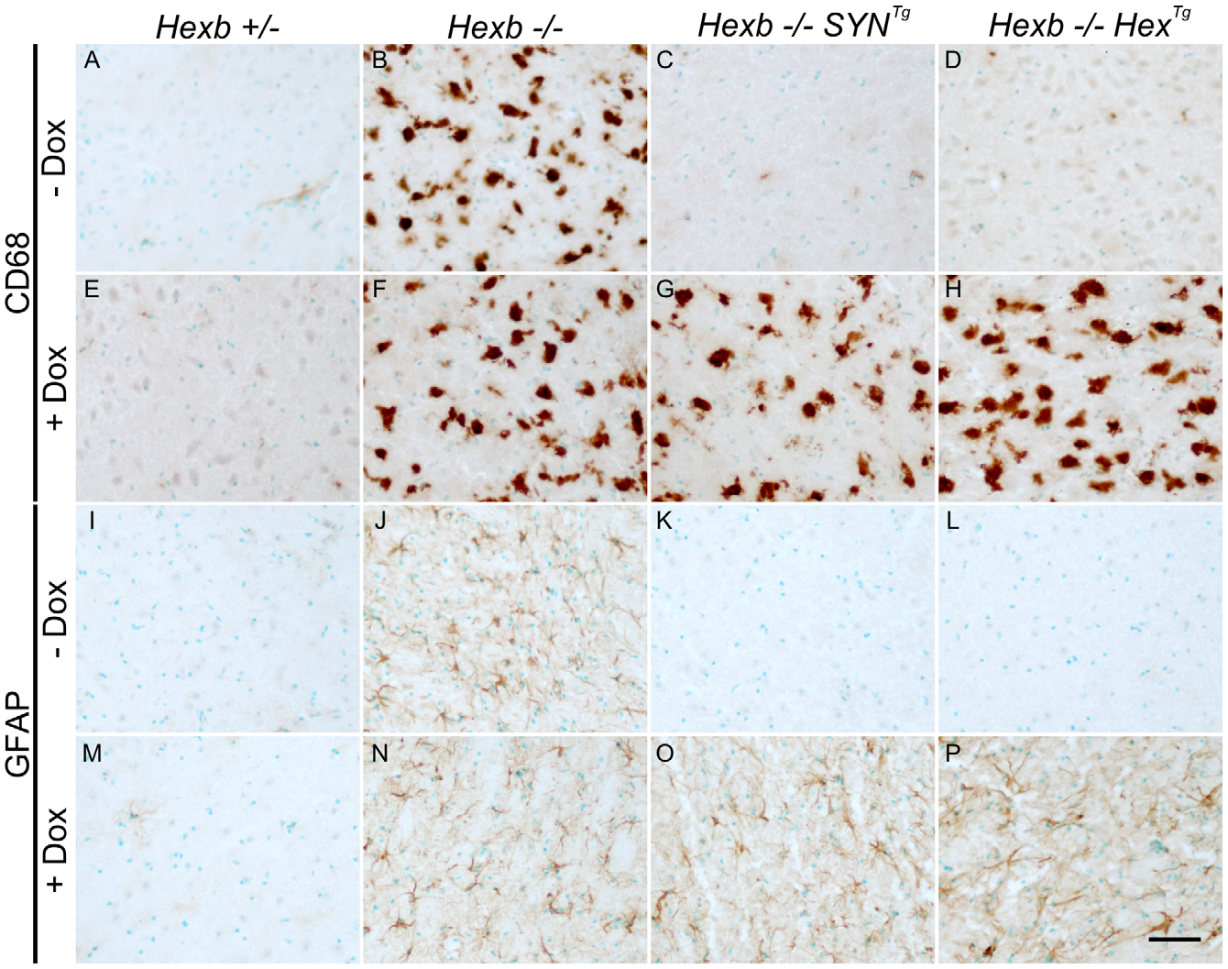
Induction of neuroinflammation by doxycycline-mediated suppression of transgenic *Hexb*. CD68 staining (brown DAB staining) shows activated microglia in the thalamus (A–H). In animals heterozygous for *Hexb* (A and E), limited CD68 staining was present. *Hexb−/−* animals (B and F) had large amoeboid microglia that stained for CD68 in the presence or absence of doxycycline (Dox). In Sandhoff animals with either the SYN or the Hex cassette, no neuroinflammation is present in the absence of doxycycline (C and D). However, in the presence of doxycycline, animals developed marked microgliosis (G and H) similar to Sandhoff animals at their humane endpoint. Comparable results were observed with GFAP staining for astrocytes (I–P). Scale bar = 50 µm.

After four to five months of exposure to doxycycline, amounts of storage material in *Hexb−/−Hex^Tg^* and *Hexb−/−SYN^Tg^* mouse brain were similar to germline *Hexb−/−* mice at their humane endpoint as shown by TLC analysis (Figure 7A and 7B). PAS staining also revealed storage neurons in doxycycline exposed *Hexb−/−Hex^Tg^* and *Hexb−/−SYN^Tg^* mice where there was no trace of PAS staining in animals not exposed to doxycycline (Figure 7C). This showed that doxycycline mediated silencing of *Hexb* expression was sufficient to cause accumulation of glycosphingolipids. Similarly, another histological hallmark of acute Sandhoff disease was also apparent in the same tissue. Staining for the neuroinflammatory markers glial fibrillary acidic protein (GFAP) and CD68 (showing activated astroglia and microglia, respectively) was markedly increased in the cerebrum and cerebellum at the humane endpoint in animals that had been exposed to doxycycline (Figure 8). Staining for neuroinflammatory markers in doxycycline exposed *Hexb−/−Hex^Tg^* and *Hexb−/−SYN^Tg^* animals appeared similar to *Hexb−/−* animals at their respective humane endpoints. Furthermore, doxycycline itself had no affect on neuroinflammation in either *Hexb+/−* or *Hexb−/−* animals (Figure 8).

## Discussion

Using autoregulatory constructs, we report the generation of inducible mouse models of Sandhoff disease. The single inducible constructs used here showed widespread expression of β-hexosaminidase in the mouse brain and rescued acute Sandhoff disease. Our inducible models displayed near-total gene silencing in the presence of doxycycline: administration of the agent induced Sandhoff disease with all its stereotypic features in the adult mouse. Moreover, expression from the transgenic constructs proved to be reversible on withdrawing the doxycycline in vivo.

The use of a single genetic construct carrying both elements (the tet-transactivator and coding sequence expressed from a tet-responsive element bearing promoter) of the tet-inducible system is not in common use for generating transgenic animals by pronuclear microinjection. This stratagem has been used to deliver autoregulatory cassettes by viral vectors [25]–[27] and to generate targeted ‘knock-ins’ in stem cells utilizing the Rosa26 locus [28], [29]. Here we show the utility of this approach is feasible for creating functional inducible transgenic mice by pronuclear injection into fertilized oocytes. The obvious advantage of using a single genetic cassette is that breeding schedules are simplified and reduced numbers of animals are required when an inducible system is bred onto a knockout background.

When crossed onto a *Hexb−/−* background, both the Hex and SYN inducible cassettes rescued the mouse from acute Sandhoff disease. However, there were differences in expression pattern between the two constructs. Although the *Hexb* promoter used to drive the Hex inducible cassette was intended to provide systemic expression of *Hexb* based on its ‘housekeeping function’, expression of β-hexosaminidase activity outside the brain was only found in the skin and skeletal muscle. This precludes assessment of the role of β-hexosaminidase activity in organs such as the liver and kidneys; however it was surprising that for each promoter, expression throughout the central nervous system was similar, since, even for the construct driven by the *Hexb* promoter, expression appeared to be confined to neurons. Lack of expression from the Hex transgene may have been due to the absence of expression elements in the construct used in this study. Alternatively, this phenomenon could be explained by position effects [19].

Although expression of transgenic β-hexosaminidase throughout the central nervous system rescued the *Hexb−/−* mouse from acute Sandhoff disease, rescue was incomplete and residual neurodegenerative disease became apparent beyond six months of age (Videos S5 and S6). At the humane endpoint, *Hexb−/−Hex^Tg^* and *Hexb−/−SYN^Tg^* mice had a mild tremor, and sporadic glycoconjugate storage was seen in Purkinje cells in the cerebellum as revealed by PAS staining; indeed, both strains showed abundant storage of glycoconjugate in lobe ten of the cerebellum. Storage was also detected in regions that interact with the cerebellum such as the pons and the red nucleus. Of note, no pathological storage was found in the substantia nigra at the delayed humane endpoint in these animals.

The most obvious aspect of residual neurological disease in the transgenic animals was increased limb tone (spasticity) - observed as clasping of the limbs when the mice were lifted by their tails. This disease feature was associated with a reproducible pattern of storage in the brain at the humane endpoint (Figure S4, Table S2). Most cerebral structures in the forebrain were free of storage material. Importantly, the motor cortex (origin of the cortico-spinal tract) showed no storage of glycolipid and the striatum (except for the globus pallidus) was also free of PAS-staining material. Accumulation of glycoconjugate was found in neurons, accompanied by CD68-positive amoeboid microglia, in the reticular nuclei in the pons and medulla and throughout the ventral spinal cord (Figure 4). Although storage did occur in other centres of the brain, such as the septum (both strains) and the hypothalamus (*Hexb−/−Hex^Tg^*), pathological changes in reticular nuclei of the medulla and pons and in ventral spinal interneurons are thought to contribute to spasticity through modulation of lower motor neurons [30]–[34]. The ventral spinal cord in *Hexb−/−Hex^Tg^* mice at the humane endpoint showed loss of PAX2-positive ventral interneuron density. Loss of PAX2-staining ventral interneurons is supported by similar results obtained by staining for the neural cell marker NeuN [35]. It is noteworthy that this result was comparable to loss of ventral interneurons in a study of a spastic mouse model [36], suggesting that loss of interneurons in the ventral spinal cord might be responsible for limb spasticity in long-surviving *Hexb−/−Hex^Tg^* mice. Similar loss of PAX2-positive interneuron density was also observed in the *Hexb−/−* animal at its endpoint, and although limb clasping was less marked, this probably reflects increasing spastic paralysis.

Declining motor function (Figure 2B) may contribute to the final deterioration of the animals, which reach the humane endpoint (Figure 2A) past one year of age. Although we cannot exclude the presence of disease in peripheral organs playing a role in weight loss, other studies from this laboratory [21], [23] showed that correction in the central nervous system was sufficient to rescue mice from acute Sandhoff disease for up to two years.

Efficient silencing of transgenic *Hexb* mRNA expression was seen in both inducible strains of mice when exposed to doxycycline (Figure 5). In the brain, transcript disappeared within 24 hours of doxycycline exposure and half life of enzyme activity was about four days, similar to human *HEXB* activity in fibroblasts that had a half life of six days [37]. In contrast, recovery of transgene expression upon withdrawal of doxycycline differed greatly between the two strains. In the frontal cortex of the Hex line, *Hexb* transgene recovered to expression levels comparable with pre-doxycycline exposure, within six days. The frontal cortex of the SYN line recovered its transgene expression much more slowly, such that 18 days after doxycycline withdrawal β-hexosaminidase activity had only recovered to approximately one third of pre-doxycycline exposure levels. Rapid recovery of transgene expression in the Hex line after doxycycline withdrawal means that it was not slow clearance of doxycycline that was causing sluggish recovery of transgene expression in the SYN line; this was considered because the skeleton can act as a reservoir of doxycycline [38]. Incomplete recovery of transgene expression in tet-inducible systems has been reported in other models [39], [40] and this is largely ascribed to changes in chromatin configuration during silencing that influence the accessibility and expression of transgenes [41], [42].

Suppression of β-hexosaminidase expression in the adult mouse induced an acute phenotype of Sandhoff disease. This was comparable in progression and phenotype to the disease seen in the germline *Hexb−/−* animal, where pathogenesis takes place alongside dynamic developmental processes. During rodent brain development, ganglioside synthesis changes dramatically. Gangliosides GM3 and GD3, which are abundant in the early fetal brain, decrease in expression and give way to increasing synthesis of GM1 and GD1a as neurons differentiate [43], [44]. Additionally, studies on developing postnatal cerebral cortex show a large temporary induction of GM2 expression that correlates tightly with strict developmental patterns of dendritogenesis [45]–[47]. Based on this observation, we predicted that after initiation in the adult mouse, progression of disease may take much longer than in the conventional germline model of Sandhoff disease. The fact that Sandhoff disease progressed largely unmodified when initiated in the adult mouse suggests that either developmental changes in ganglioside synthesis are insignificant compared with adult neuronal output of ganglioside, or that lysosomal disorder disease processes are resilient to fluctuations in the absolute amount of storage material. Absence of a developmental component of neurological pathogenesis has also been observed in a conditional model of Niemann-Pick type C disease [48] and may be a general feature of neuronopathic lysosomal disorders.

In the present study, a high dose of doxycycline was used to completely abolish transgene expression. However, it may be possible to titrate the dose of doxycycline to give partial correction of the phenotype - as has been reported in studies of GDNF expression induced by adeno-associated viral vectors under the control of the tet system in the substantia nigra [49]. By allowing partial rescue of the Sandhoff phenotype mediated by either of the transgenes, this approach might be used to generate attenuated models of GM2 gangliosidosis.

We contend that the inducible models of Sandhoff disease reported here will facilitate exploration of the pathogenesis in GM2 gangliosidosis. In mice that were allowed to develop residual elements of Sandhoff disease, regionalized storage of glycolipids and related storage molecules in the brain showed that limb hypertonicity can arise independently from glycolipid storage in the motor cortex and could originate in the hindbrain and spinal cord. Tetracycline-inducible models of acute Sandhoff disease were responsive to doxycycline and showed that the pathogenesis of acute Sandhoff disease is not dependent or significantly modified by processes that occur in the developing brain. Future research will focus on the effects of increasing transgene dosage; in addition, the inducible models will be useful for identifying early cellular events in the evolution of experimental GM2 gangliosidosis in vivo.

## Supporting Information

Figure S1 In vitro validation of inducible expression constructs. To determine whether cassettes would express *Hexb* coding sequence, HEK 293T cells were transiently transfected using the calcium phosphate method and six micrograms of plasmid DNA. One day after cells were transfected, total β-hexosaminidase activity was assessed in cell culture media using the MUG assay. Background controls included *Hexb* cDNA under control of the tet-response element without the tet-transactivator, and GFP driven from a CMV promoter. A number of versions of the Hex and SYN inducible constructs were tested in parallel (shown in the figure legend) in the absence and in the presence of varying concentrations of doxycycline. The Hex and SYN constructs outlined by a red box were selected for microinjection into mouse embryos. BGH PA = bovine growth hormone poly adenylation signal, cHS4 = chicken hyper sensitive region 4 insulator fragment (+/−refers to opposite orientations), Hex = mouse *Hexb* promoter, Hexb = *Hexb* coding sequence, SV40 PA = simian virus 40 poly adenylation signal, SYN1 = human *SYN1* promoter, TRE-CMVΔ = tet responsive element – cytomegalovirus minimal promoter (TRE-Tight), tTA2^s^ = tet-transactivator coding sequence (tet-off). Bars indicate mean of triplicate β-hexosaminidase activity measurements from single transfections.
(TIF)

Figure S2 Separation of β-hexosaminidase isoforms by anion-exchange chromatography. Samples of cerebrum were homogenized and separated by anion-exchange chromatography using a resource Q column. The first three fractions collected (column flow-through) were 1 ml each. Fractions collected during elution with a rising concentration of NaCl (100 mM–400 mM) were 0.5 ml each. The y-axis shows β-hexosaminidase activity assessed with the MUG assay as nmoles cleaved per hour (per assay well with 10 µl of fraction added). Values were divided by mg of protein loaded onto the ion exchange column. Fractions containing activity from different β-hexosaminidase isozymes are coloured as follows: HexB = red, HexA = blue, HexS = green. A shows the expected pattern of enzyme activity from a *Hexb+/−* animal as all isozymes are present. B shows a Sandhoff animal (*Hexb−/−*) that only possessed the HexS isozyme (β-hexosaminidase α/α homodimer), shown as a peak in the green fractions. C and E show *Hexb−/−* animals that also have the Hex or SYN transgenic *Hexb* expression cassettes, respectively. These animals, like the *Hexb+/−* mouse, also have all three β-hexosaminidase isoforms. After exposure to dietary doxycycline (for between four and five months, in this case), transgenic *Hexb* expression was suppressed and HexB and HexA isoforms were no longer present in the cerebrum (D and F).
(TIF)

Figure S3 In situ hybridization showing tet-transactivator mRNA in the brain and staining for β-hexosaminidase activity. Staining for tet-transactivator transcript (purple NBT/BCIP staining) correlated with staining for transgenic β-hexosaminidase activity (red), except for the olfactory bulbs of the *Hexb−/−SYN^Tg^* mouse, where transcript could not be found in spite of intense β-hexosaminidase activity. Scale bar = 500 µm, except for the cerebellar cortex where scale bar = 200 µm.
(TIF)

Figure S4 Glycoconjugate storage in the rescued Sandhoff mouse at humane endpoint. Rescued Sandhoff mice (*Hexb−/−Hex^Tg^* or *Hexb−/−SYN^Tg^*) in the absence of doxycycline developed residual storage of glycoconjugates, revealed with PAS staining (symbolized as red dots on the pictograms). The *Hexb−/−Hex^Tg^* mouse had more residual storage in the ventral forebrain (E–K) and the midbrain (L–O) than the *Hexb−/−SYN^Tg^* mouse. However, rescue from storage was more complete in the *Hexb−/−Hex^Tg^* mouse strain cerebellum than in the *Hexb−/−SYN^Tg^* mouse strain (P–U).
(TIF)

Figure S5 Weight loss in doxycycline-inducible Sandhoff animals. (A and B) *Hexb−/−Hex^Tg^* and *Hexb−/−SYN^Tg^* animals, respectively, put on weight steadily when fed normal lab chow (blue diamonds). When animals were fed a doxycycline laced diet from five weeks of age onward, mouse weight reached a plateau between 15 and 20 weeks of age and declined to humane endpoint within the next five to six weeks (n = 6, sex matched). (C and D) There appeared to be no difference in weight gain between *Hexb−/−Hex^Tg^* and *Hexb−/−SYN^Tg^* animals fed normal lab chow (blue diamonds) and *Hexb+/−*healthy controls fed doxycycline laced diet (green triangles) (n = 5, sex matched, for all groups). Data points represent mean ± SEM.
(TIF)

Table S1 Production of transgenic founder mice. F2 B6CBA fertilized oocytes were microinjected with Hex or SYN constructs, outlined in red in Figure S1. Of the embryos that survived microinjection and implantation into pseudopregnant females, 10–20% of live births produced transgenic founders that had integrated the transgenic construct indicated above. Once separate transgenic lines had been crossed from a *Hexb+/+* onto a *Hexb−/−* background, a total of two transgenic lines were found to express transgenic *Hexb* in the central nervous system (CNS), as detected by Hex activity staining (Figure 1C).
(DOC)

Table S2 Storage of glycoconjugate shown by PAS staining in *Hexb−/−Hex^Tg^* and *Hexb−/−SYN^Tg^* brain at humane endpoint. − = no PAS staining, +++ = strong/widespread PAS staining similar to *Hexb−/−* mouse at humane endpoint.
(DOC)

Video S1 *Hexb+/−* mouse, six months of age.
(WMV)

Video S2 *Hexb−/−* mouse, humane endpoint.
(WMV)

Video S3 *Hexb−/−Hex^Tg^* mouse (−dox), six months of age.
(WMV)

Video S4 *Hexb−/−SYN^Tg^* mouse (−dox), six months of age.
(WMV)

Video S5 *Hexb−/−Hex^Tg^* mouse (−dox), one year of age.
(WMV)

Video S6 *Hexb−/−SYN^Tg^* mouse (−dox), one year of age.
(WMV)

Video S7 *Hexb−/−Hex^Tg^* mouse fed doxycycline starting at five weeks of age, viewed at humane endpoint of approximately six months of age.
(WMV)

Video S8 *Hexb−/−SYN^Tg^* mouse fed doxycycline starting at five weeks of age, viewed at humane endpoint of approximately six months of age.
(WMV)
