# Supplementary figures and images for: Characterization of Inducible Models of Tay-Sachs and Related Disease
Source: PLoS Genet. 2012 Sep 20;8(9):e1002943. doi: 10.1371/journal.pgen.1002943 (PMC3447966; doi:10.1371/journal.pgen.1002943)

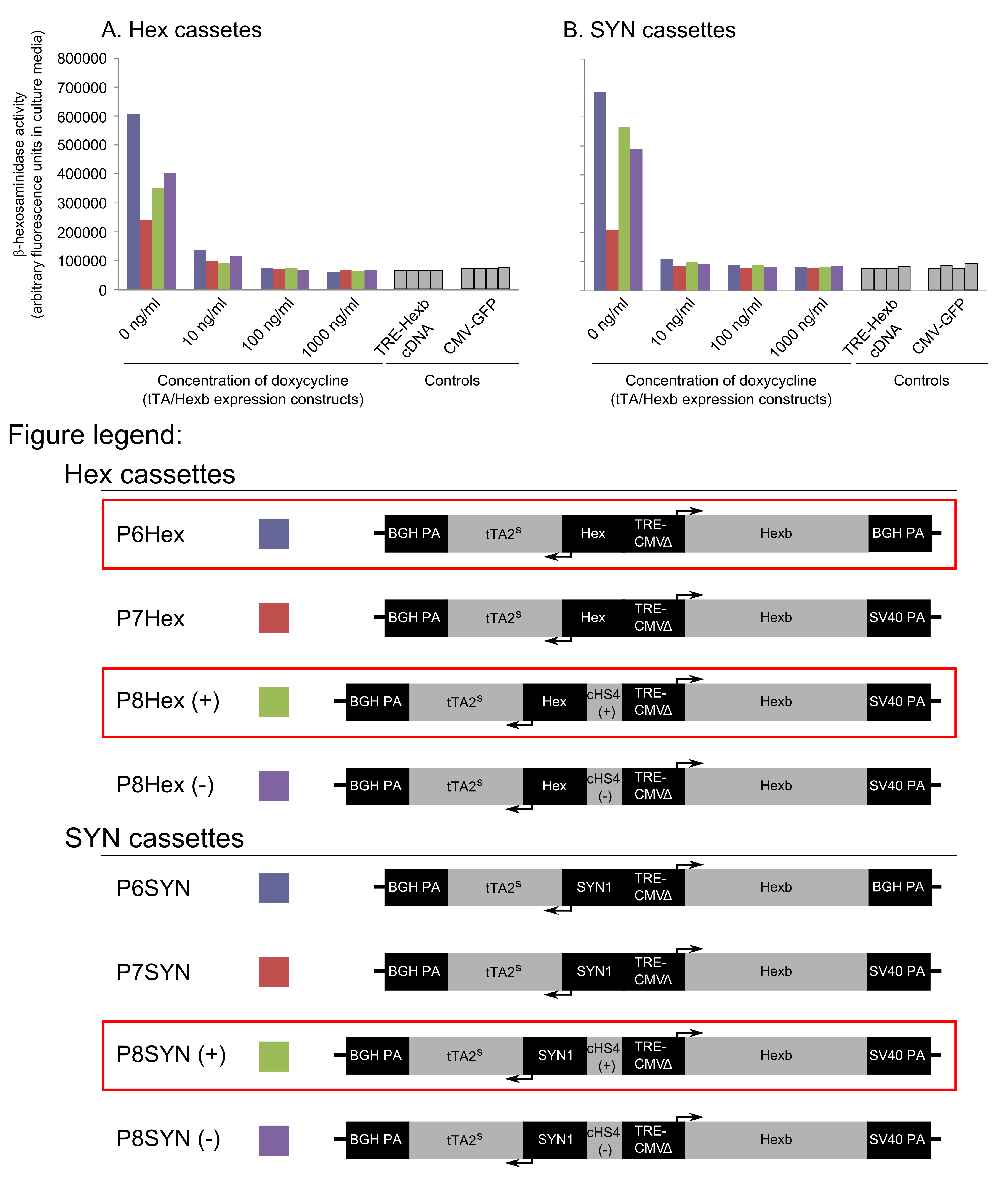

Supplement: Figure S1 — In vitro validation of inducible expression constructs. To determine whether cassettes would express Hexb coding sequence, HEK 293T cells were transiently transfected using the calcium phosphate method and six micrograms of plasmid DNA. One day after cells were transfected, total β-hexosaminidase activity was assessed in cell culture media using the MUG assay. Background controls included Hexb cDNA under control of the tet-response element without the tet-transactivator, and GFP driven from a CMV promoter. A number of versions of the Hex and SYN inducible constructs were tested in parallel (shown in the figure legend) in the absence and in the presence of varying concentrations of doxycycline. The Hex and SYN constructs outlined by a red box were selected for microinjection into mouse embryos. BGH PA = bovine growth hormone poly adenylation signal, cHS4 = chicken hyper sensitive region 4 insulator fragment (+/−refers to opposite orientations), Hex = mouse Hexb promoter, Hexb = Hexb coding sequence, SV40 PA = simian virus 40 poly adenylation signal, SYN1 = human SYN1 promoter, TRE-CMVΔ = tet responsive element – cytomegalovirus minimal promoter (TRE-Tight), tTA2s = tet-transactivator coding sequence (tet-off). Bars indicate mean of triplicate β-hexosaminidase activity measurements from single transfections. (TIF) [file pgen.1002943.s001.tif]

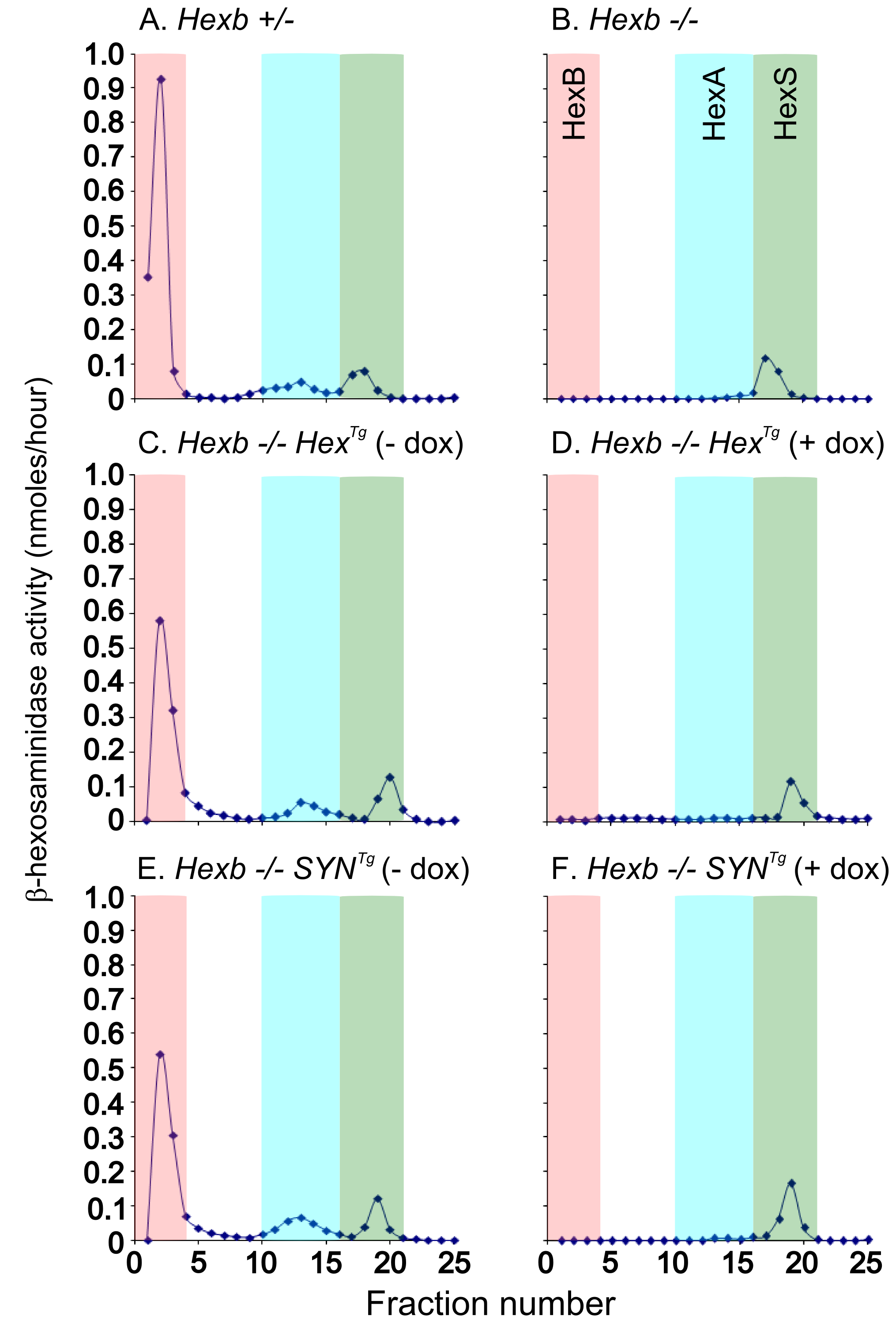

Supplement: Figure S2 — Separation of β-hexosaminidase isoforms by anion-exchange chromatography. Samples of cerebrum were homogenized and separated by anion-exchange chromatography using a resource Q column. The first three fractions collected (column flow-through) were 1 ml each. Fractions collected during elution with a rising concentration of NaCl (100 mM–400 mM) were 0.5 ml each. The y-axis shows β-hexosaminidase activity assessed with the MUG assay as nmoles cleaved per hour (per assay well with 10 µl of fraction added). Values were divided by mg of protein loaded onto the ion exchange column. Fractions containing activity from different β-hexosaminidase isozymes are coloured as follows: HexB = red, HexA = blue, HexS = green. A shows the expected pattern of enzyme activity from a Hexb+/− animal as all isozymes are present. B shows a Sandhoff animal (Hexb−/−) that only possessed the HexS isozyme (β-hexosaminidase α/α homodimer), shown as a peak in the green fractions. C and E show Hexb−/− animals that also have the Hex or SYN transgenic Hexb expression cassettes, respectively. These animals, like the Hexb+/− mouse, also have all three β-hexosaminidase isoforms. After exposure to dietary doxycycline (for between four and five months, in this case), transgenic Hexb expression was suppressed and HexB and HexA isoforms were no longer present in the cerebrum (D and F). (TIF) [file pgen.1002943.s002.tif]

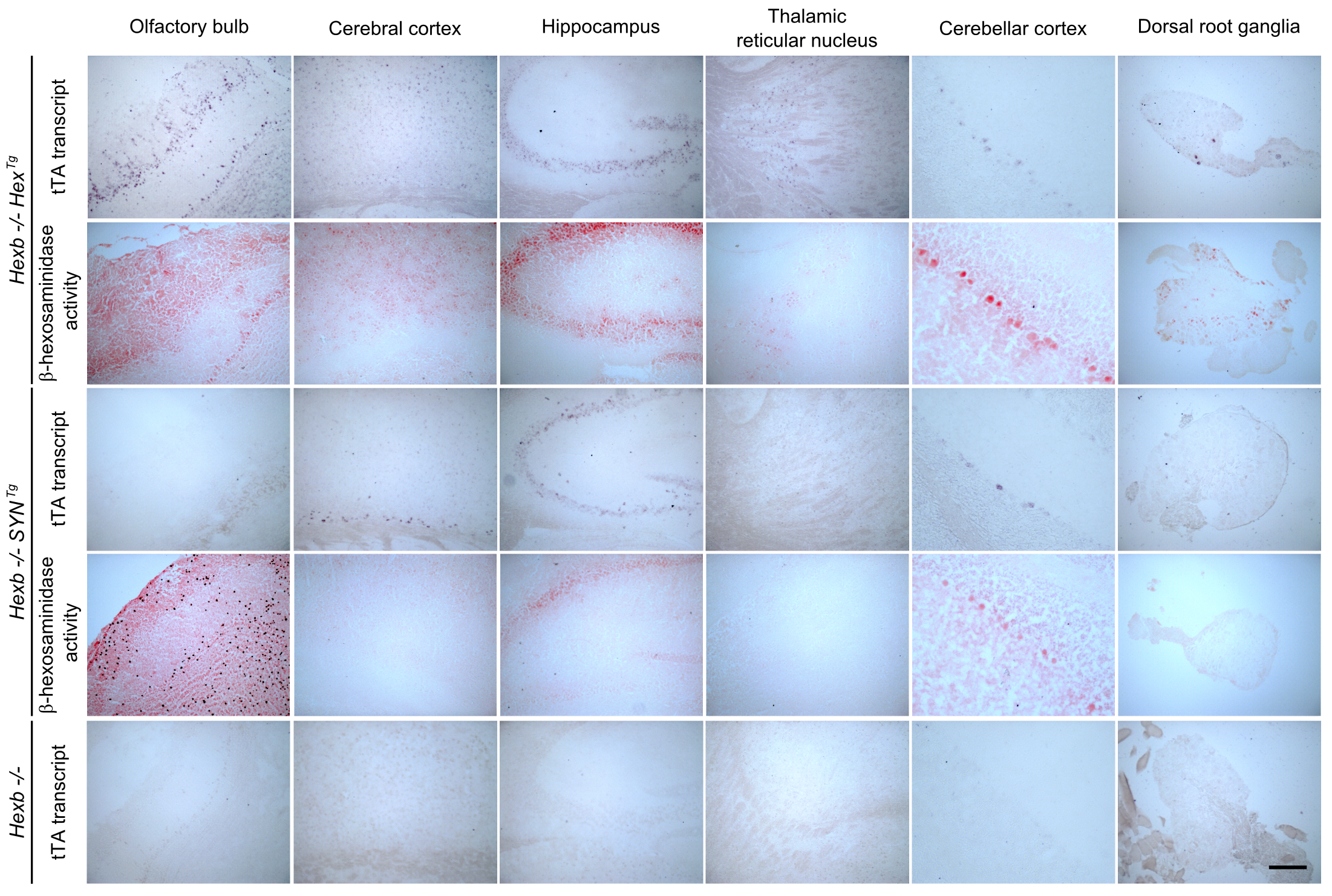

Supplement: Figure S3 — In situ hybridization showing tet-transactivator mRNA in the brain and staining for β-hexosaminidase activity. Staining for tet-transactivator transcript (purple NBT/BCIP staining) correlated with staining for transgenic β-hexosaminidase activity (red), except for the olfactory bulbs of the Hexb−/−SYNTg mouse, where transcript could not be found in spite of intense β-hexosaminidase activity. Scale bar = 500 µm, except for the cerebellar cortex where scale bar = 200 µm. (TIF) [file pgen.1002943.s003.tif]

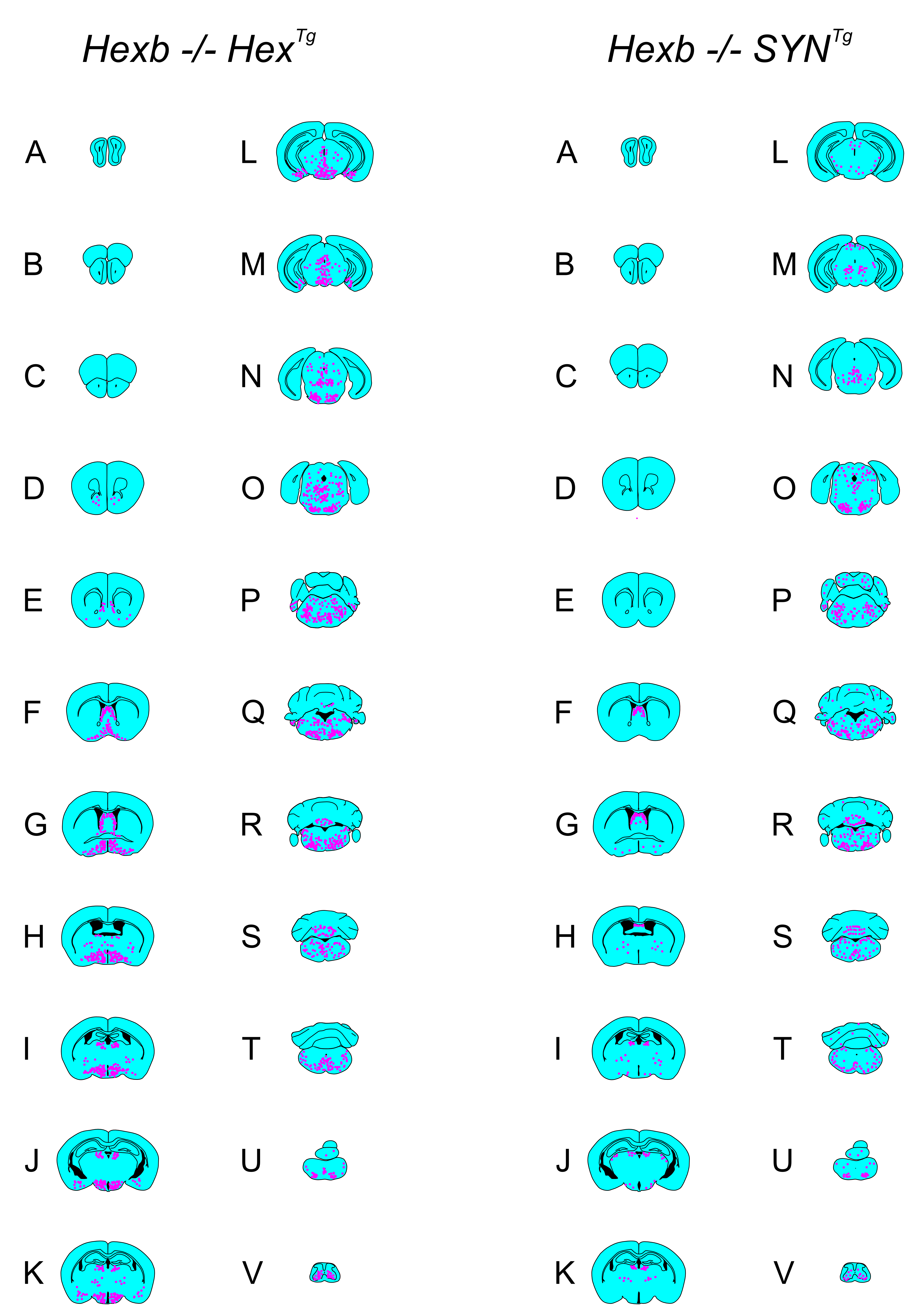

Supplement: Figure S4 — Glycoconjugate storage in the rescued Sandhoff mouse at humane endpoint. Rescued Sandhoff mice (Hexb−/−HexTg or Hexb−/−SYNTg) in the absence of doxycycline developed residual storage of glycoconjugates, revealed with PAS staining (symbolized as red dots on the pictograms). The Hexb−/−HexTg mouse had more residual storage in the ventral forebrain (E–K) and the midbrain (L–O) than the Hexb−/−SYNTg mouse. However, rescue from storage was more complete in the Hexb−/−HexTg mouse strain cerebellum than in the Hexb−/−SYNTg mouse strain (P–U). (TIF) [file pgen.1002943.s004.tif]

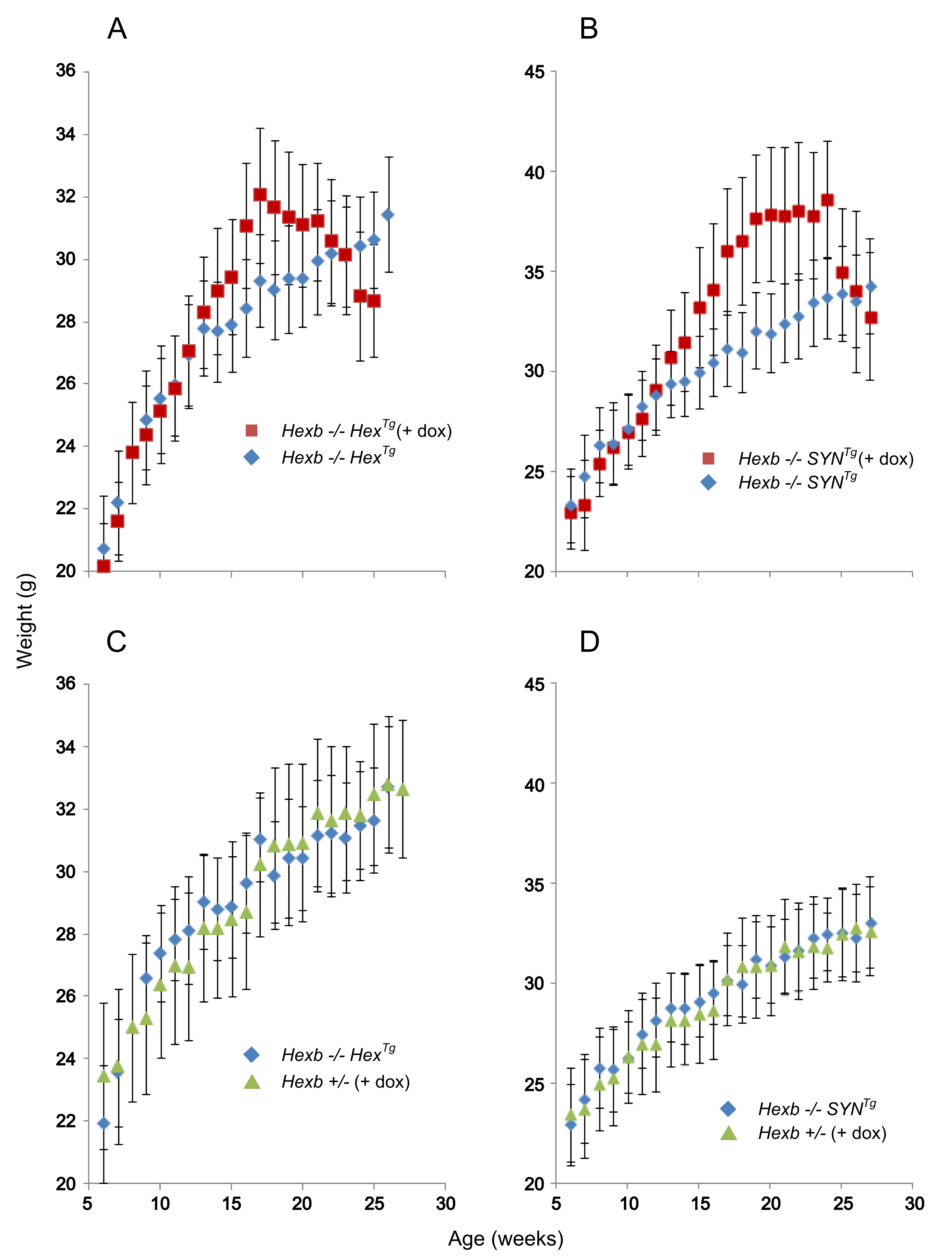

Supplement: Figure S5 — Weight loss in doxycycline-inducible Sandhoff animals. (A and B) Hexb−/−HexTg and Hexb−/−SYNTg animals, respectively, put on weight steadily when fed normal lab chow (blue diamonds). When animals were fed a doxycycline laced diet from five weeks of age onward, mouse weight reached a plateau between 15 and 20 weeks of age and declined to humane endpoint within the next five to six weeks (n = 6, sex matched). (C and D) There appeared to be no difference in weight gain between Hexb−/−HexTg and Hexb−/−SYNTg animals fed normal lab chow (blue diamonds) and Hexb+/−healthy controls fed doxycycline laced diet (green triangles) (n = 5, sex matched, for all groups). Data points represent mean ± SEM. (TIF) [file pgen.1002943.s005.tif]
