# Supplementary material for: Characterization of Inducible Models of Tay-Sachs and Related Disease
Source: PLoS Genet. 2012 Sep 20;8(9):e1002943. doi: 10.1371/journal.pgen.1002943 (PMC3447966; doi:10.1371/journal.pgen.1002943)
Supplement: Table S1 — Production of transgenic founder mice. F2 B6CBA fertilized oocytes were microinjected with Hex or SYN constructs, outlined in red in Figure S1. Of the embryos that survived microinjection and implantation into pseudopregnant females, 10–20% of live births produced transgenic founders that had integrated the transgenic construct indicated above. Once separate transgenic lines had been crossed from a Hexb+/+ onto a Hexb−/− background, a total of two transgenic lines were found to express transgenic Hexb in the central nervous system (CNS), as detected by Hex activity staining (Figure 1C). (DOC) [file pgen.1002943.s006.doc]

| Construct | P8Hex (+) | P8SYN (+) | P6Hex |
| --- | --- | --- | --- |
| Live births from microinjected embryos | 45 | 35 | 23 |
| Number of transgenic integrants | 9 | 4 | 3 |
| Transgenic integrants that expressed *Hexb* coding sequence in the CNS | 1 | 1 | 0 |
