# Supplementary material for: Characterization of Inducible Models of Tay-Sachs and Related Disease
Source: PLoS Genet. 2012 Sep 20;8(9):e1002943. doi: 10.1371/journal.pgen.1002943 (PMC3447966; doi:10.1371/journal.pgen.1002943)
Supplement: Table S2 — Storage of glycoconjugate shown by PAS staining in Hexb−/−HexTg and Hexb−/−SYNTg brain at humane endpoint. − = no PAS staining, +++ = strong/widespread PAS staining similar to Hexb−/− mouse at humane endpoint. (DOC) [file pgen.1002943.s007.doc]

| Region | *Hexb -/- Hex^Tg^* | *Hexb -/-* *SYN^Tg^* |
| --- | --- | --- |
| Olfactory bulb |  |  |
| Granule cell layer | - | - |
| Mitral cell layer | - | - |
| Glomerular cell layer | - | - |
| Anterior olfactory nucleus | - | - |
| Accessory olfactory bulb | - | - |
| Cerebrum |  |  |
| Primary and secondary motor cortex | - | - |
| Primary and secondary somatosensory cortex | - | - |
| Piriform cortex | - | - |
| Agranular insular cortex | - | - |
| Gustatory cortex | - | - |
| Anterior cingulate cortex | - | - |
| Retrosplenial cortex | - | - |
| Entorhinal cortex | - | - |
| Auditory cortex | - | - |
| Parietal association cortex | - | - |
| Visual cortex | - | - |
| Temporal association cortex | - | - |
| CA1 field | - | - |
| CA3 field | - (Ventral CA3 ++) | - |
| Dentate gyrus | - | - |
| Amygdalo-hippocampal area | ++/+++ | - |
| Nucleus accumbens | - | - |
| Olfactory tubercle | - | - |
| Caudate putamen | - | - |
| Globus pallidus | +/++ | +/++ |
| Substantia innominata | +/++ | - |
| Preoptic nuclei | +++ | -/+ |
| Nucleus of the diagonal band of Broca | +++ | - |
| Lateral septum | +++ | ++ |
| Medial septum | + | - |
| Amygdaloid nuclei | - | - |
| Anterior thalamic nuclei | - (Lateral habenula +++, laterodorsal nucleus +) | - (Lateral habenula ++) |
| Posterior thalamic nuclei | - (Posterior nucleus ++/+++) | - (Posterior nucleus ++/+++, ventrobasal nuclei +) |
| Thalamic reticular nucleus | - | -/+ |
| Anterior hypothalamus | +++ | - |
| Lateral hypothalamus | +++ | +/- |
| Medial hypothalamus | +++ | +/- |
| Posterior hypothalamus | +++ | - |
| Midbrain |  |  |
| Periaqueductal grey | (Rostral ++, caudal -) | - |
| Superior colliculus | - (Deep grey layer of, ++) | - |
| Mesencephalic reticular nucleus | +++ | + |
| Substantia nigra | - | - |
| Ventral tegmental area | +/++ | -/+ |
| Pontine grey | +/++ | + |
| Medial geniculate nucleus | - | + |
| Red nucleus | +++ | ++ |
| Pontine reticular nucleus | +++ | ++/+++ |
| Medial midbrain nuclei | ++ | - |
| Inferior colliculus | - | + |
| Mammillary nucleus | +++ | - (Supramammillary nucleus +++) |
| Hindbrain |  |  |
| Motor V | +++ | ++ |
| Sensory V | +/++ | +/++ |
| Cochlear nucleus | +/++ | +/++ |
| Motor VII | +++ | ++ |
| Parvocellular reticular nucleus | ++ | + |
| Intermediate reticular nucleus | ++ | + |
| Gigantocellular reticular nucleus | +++ | ++ |
| Magnocellular reticular nucleus | +++ | ++/+++ |
| Vestibular nuclei | + | + |
| Superior olivary complex | -/+ | + |
| Inferior olivary complex | - (Principal nucleus of the olivary complex ++) | + |
| Nucleus of the solitary tract | - | (Rostral ++, caudal -) |
| Cuneate nucleus | ++ | ++/+++ |
| Spinal nucleus of the trigeminal nerve | + | +/++ |
| Motor XII | +++ | + |
| Cerebellum |  |  |
| Anterior lobe | - | -/+ |
| Paraflocculus | -/+ | -/+ |
| Flocculus | +/++ | -/+ |
| Central lobe 3 | - | -/+ |
| Central lobe 2 | - | -/+ |
| Simple lobule | - | -/+ |
| Anisoform lobe 1 | -/+ | -/+ |
| Central lobe 4 | - | -/+ |
| Central lobe 5 | -/+ | -/+ |
| Central lobe 1 | -/+ | -/+ |
| Anisoform lobe 2 | - | -/+ |
| Central lobe 6 | -/+ | -/+ |
| Central lobe 10 | +++ | +++ |
| Central lobe 9 | ++ | ++ |
| Paramedian lobe | -/+ | - |
| Copula Pyramidis | -/+ | - |
| Central lobe 7 | - | - |
| Fastigial nucleus | - | - |
| Nucleus interpositus | - | - |
| Dentate nucleus | - | - |
| Spinal cord |  |  |
| Dorsal grey matter (I-V) | + | + |
| Ventral grey matter (VII-X) | ++/+++ | ++ |
